# Privileged Quinolylnitrones for the Combined Therapy of Ischemic Stroke and Alzheimer’s Disease

**DOI:** 10.3390/ph14090861

**Published:** 2021-08-27

**Authors:** José M. Alonso, Alejandro Escobar-Peso, Alejandra Palomino-Antolín, Daniel Diez-Iriepa, Mourad Chioua, Emma Martínez-Alonso, Isabel Iriepa, Javier Egea, Alberto Alcázar, José Marco-Contelles

**Affiliations:** 1 Laboratory of Medicinal Chemistry (IQOG, CSIC), Juan de la Cierva 3, 28006 Madrid, Spain; xosemag@yahoo.es (J.M.A.); daniel.diezi@uah.es (D.D.-I.); mchioua@gmail.com (M.C.); 2 Department of Research, IRYCIS, Hospital Ramón y Cajal, Ctra. Colmenar Km 9.1, 28034 Madrid, Spain; alejandro.escobar@hrc.es (A.E.-P.); emma.martinez@hrc.es (E.M.-A.); 3 Molecular Neuroinflammation and Neuronal Plasticity Research Laboratory, Research Unit, Hospital Universitario Santa Cristina, Instituto de Investigación Sanitaria-Hospital Universitario de la Princesa, 28009 Madrid, Spain; apantolin@gmail.com; 4 Departamento de Química Orgánica y Química Inorgánica, Universidad de Alcalá, Ctra. Madrid-Barcelona Km 33.6, 28871 Alcalá de Henares, Spain; isabel.iriepa@uah.es; 5 Institute of Chemical Research Andrés M. del Río, Alcalá University, 28805 Alcalá de Henares, Spain

**Keywords:** Alzheimer’s disease, ischemic stroke, multipotent drugs, neuroprotection, quinolylnitrones

## Abstract

Cerebrovascular diseases such as ischemic stroke are known to exacerbate dementia caused by neurodegenerative pathologies such as Alzheimer’s disease (AD). Besides, the increasing number of patients surviving stroke makes it necessary to treat the co-occurrence of these two diseases with a single and combined therapy. For the development of new dual therapeutic agents, eight hybrid quinolylnitrones have been designed and synthesized by the juxtaposition of selected pharmacophores from our most advanced lead-compounds for ischemic stroke and AD treatment. Biological analyses looking for efficient neuroprotective effects in suitable phenotypic assays led us to identify MC903 as a new small quinolylnitrone for the potential dual therapy of stroke and AD, showing strong neuroprotection on (i) primary cortical neurons under oxygen–glucose deprivation/normoglycemic reoxygenation as an experimental ischemia model; (ii), neuronal line cells treated with rotenone/oligomycin A, okadaic acid or β-amyloid peptide A*β*_25–35_, modeling toxic insults found among the effects of AD.

## 1. Introduction

Due to the high quality of life in our developed countries, and the increased life expectancy of the eldest, ageing diseases such as stroke and Alzheimer’s disease (AD) have become first order individual, socio-economic and medical problems with unmet and limited therapies [1]. Consequently, the search for new and more efficient therapies for stroke and AD is urgent and one of the most active areas of research in the current neuroscience domain [2].

**Figure 1:**
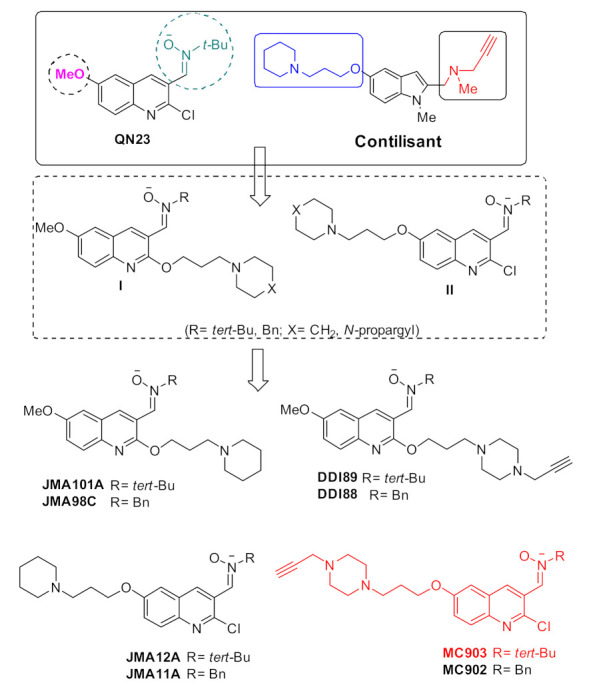
Structure of QN23, Contilisant and the new QNs described in this work.

Furthermore, the interconnection and co-occurrence of stroke and AD in the same patients is known and has been largely documented [3], although the implicated biological mechanisms have not yet been clearly established [4]. In fact, cerebrovascular diseases are not only the basis of a vascular-related cognitive impairment, referred to as vascular dementia (VD), but they are also known to exacerbate dementia caused by other factors such as AD and other degenerative diseases [5]. As noted recently by Macrae and Allan, *“…With increasing numbers of patients surviving stroke, there is also a need to focus more on post-stroke complications that affect quality of life. These include not only motor and speech impairments, but also depression, dementia, epilepsy and anxiety, among other things…”* [6]. Hence, the identification of new drugs for the combined therapy of both ischemic stroke and AD would be an ideal therapeutic issue seldom put in practice, but that currently concentrates our most recent efforts, taking advantage of two of our most advanced lead-compounds. Recently, we identified (Z)-N-tert-butyl-1-(2-chloro-6-methoxyquinolin-3-yl)-methanimine oxide (QN23) (Figure 1) as a very effective neuroprotective quinolylnitrone (QN), showing neuroprotection induction in two in vivo models of global and focal cerebral ischemia [7], resulting in a new lead-compound for ischemic stroke treatment. On the other hand, we have described that Contilisant (Figure 1) is a permeable and a highly neuroprotective agent against a number of AD-relevant toxic insults, showing in vitro inhibitory properties for neurotransmitter-catabolizing enzymes (ChE, MAO) [8] or G protein-coupled receptors (H3R [8], S1R [9]), and in vivo better protective effect than our previous hit-compound ASS234 [10] or donepezil on the Y-maze and radial arm maze task against cognitive decline induced by the *β*-amyloid peptide A*β*_1__−42_ oligomers [9]. Remarkably, QN23 and Contilisant are also potent antioxidants, a beneficial feature for the potential treatment of ischemic stroke and AD taking into account the noxious effect that oxidative stress and reactive oxygen species (ROS) exert on both pathologies [11].

With this knowledge, we have designed the new *N-tert*-butyl and *N*-benzyl hybrid QNs of types **I** and **II** shown in Figure 1, as different combinations of those privileged structural features present in QN23 and Contilisant over the QN core. QNs of type I, JMA98C and JMA101A, are the result of the insertion of the key piperidinopropyloxy motif—a polyvalent pharmacophore present in Contilisant for the effective ChE, H3R, and S1R binding [8,9]—at C2 of QN23, keeping the methoxy group at C6, a feature that seems critical for efficient neuroprotection [7]. An analogous analysis resulted in QNs DDI88 and DDI89, where we have installed an additional *N*-propargyl motif—a typical MAO pharmacophore—in a piperazinopropyloxy group—a proven pharmacophore for ChE inhibition [10]. A similar analysis has resulted in four more QNs of type **II**, such as JMA11A, JMA12A, MC902 and MC903, where we have kept the chlorine atom at C2 as in QN23, and the functionalized cycloalkylaminopropyloxy moiety linked at C6 instead of the methoxy group. Based on these designs, which maintain the structural features responsible for the neurotransmitter level regulation impaired in AD [12], on a QN core, the QNs reported here are expected to act as multipotent small molecules potentially suitable for the treatment of AD and ischemic stroke [13].

Thus, prompted by the recent communication by Sun et al. [14] on the pharmacological profile of the neuroimmunomodulator agent AD110 for the therapeutic management for AD and stroke, and the results reported by Liu et al. [15] for MT-20R—a substituted α-phenyl-*tert*-butyl nitrone bearing MAO and ChE pharmacophores for Parkinson’s disease—we describe here our preliminary results on this area. We have identified MC903 as a new small QN for the potential combined therapy of ischemic stroke and AD, showing strong neuroprotective properties on primary cortical neurons under hypoxia/reoxygenation as an experimental ischemia model, and on neuronal line cells treated with rotenone plus oligomycin A, okadaic acid or A*β*_25–35_, modeling three toxic insults found in AD.

## 2. Results and Discussion

### 2.1. Synthesis

The synthesis of the target QNs was carried out starting from readily available precursors in short synthetic sequences, affording the desired ligands as stereochemically single *Z*-isomers in pure form, good overall yields and multigram amounts (Supplementary Material). Next, the QNs were submitted to biological analysis in order to determine their neuroprotective capacity.

### 2.2. Evaluation of Neuroprotection in an Experimental Model of Ischemia in Primary Neuronal Cultures

**Figure 2:**
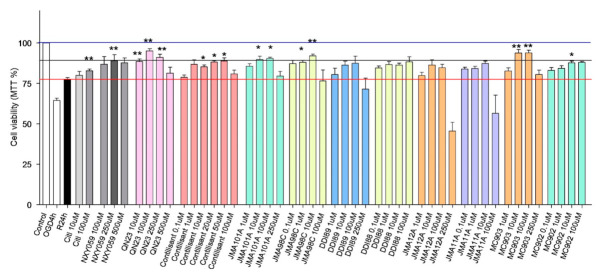
Effect of QNs JMA101A, JMA98C, DDI89, DDI88, JMA12A, JMA11A, MC903 and MC902 on primary neuronal cultures subjected to oxygen–glucose deprivation (OGD) and subsequent reoxygenation. Bar chart showing the percentage of cell viability at 24 h of normoxic recovery after OGD for 4 h either untreated (R24h) or treated with different concentrations (µM) of citicoline, NXY-059, QN23, Contilisant or other nitrones. The value induced by OGD at 4 h without recovery period (OGD4h) is also indicated. Cell viability corresponding to control cells (1.916 ± 0.07 AU) was considered as 100% (blue line). Bars represent the average of four to eight independent experiments; error bars representing the SE. * *p* < 0.05, and ** *p* < 0.01 compared with R24h (red line) by Dunnett’s post-test after ANOVA, when it was significant. Black line shows the increase of cell viability induced by the reference nitrone NXY-059 (250 µM). Statistical significances below R24h value were not shown.

QNs JMA101A, JMA98C, DDI89, DDI88, JMA12A, JMA11A, MC903 and MC902 were tested in an experimental model of ischemia in order to evaluate their potential neuroprotective effect after an ischemic insult. To do so, primary neuronal cultures were subjected to oxygen and glucose deprivation (OGD) conditions for 4 h and treated at the onset of the reoxygenation period with the previously mentioned QNs, or reference compounds such as citicoline, NXY-059, QN23 or Contilisant. Neuroprotection was monitored by the measurement of cell viability, as determined by the MTT assay (Figure 2), in which control group was set as 100%. Unrecovered experimental group (OGD4h) showed a severe decrease in cell viability (64.4 ± 1.4%, *p* < 0.0001 compared with 100% control, one-sample *t*-Test), which was only partially reversed after reoxygenation for 24 h (R24h; 77.2 ± 1.3%, *p* < 0.0001, by Student’s *t*-Test). 

The addition of 100 µM citicoline, a well-known neuroprotective agent, prompted a significant increase in cell viability (82.8 ± 1.1%) compared with untreated (vehicle) R24h group (Figure 1). When standards NXY-059 [7], QN23 [7] or Contilisant [8] were added instead of citicoline, higher levels of cell viability were achieved (88.9 ± 3.7%, 95.1 ± 1.4% and 88.7 ± 1.9% for 250 µM NXY-059, 100 µM QN23 and 50 µM Contilisant, respectively) (Figure 2). Treatment with the new QNs provided different effects on the recovery of cell metabolism after the OGD insult. In the first place, and remarkably, almost the complete set of QNs provided a higher cell viability than untreated R24h cells in the whole concentration range tested (0.1–250 µM). Based on our previous experience, we initially explored the concentration range 1–250 µM, which was expanded to 0.1 µM in those cases still showing a good response at low concentrations. Compounds DDI88 and MC902 were not assayed at 250 µM despite exerting relative good cell viability values because of solubility issues. Only QNs DDI89, JMA12A and JMA11A exerted a toxic effect—i.e., a lower cell viability than the untreated group, R24h—at the highest tested concentration of 250 µM. Best-performing QNs JMA101A at 10 and 100 µM, JMA98C at 10 µM and MC903 at 10 µM and 100 µM, achieved higher cell viability than citicoline (100 µM), reference nitrone NXY-059 (250 µM) and Contilisant (10–50 µM). Remarkably, MC903 effect at 10 µM and 100 µM (93.7 ± 2.1% and 93.8 ± 1.7%, respectively) reached values similar to the ones observed for QN23 (100 µM), our most potent QN found to date [7] (Figure 2). 

From the previous results of cell viability, we defined neuroprotection activity as the effect that achieved a cellular viability higher than the one produced by the normoxic recovery alone, determined by the untreated R24h group, which was set as 0%. Cell viability of the control group was set as 100% of neuroprotection. Neuroprotection induced by QNs JMA101A, JMA98C, DDI89, DDI88, JMA12A, JMA11A, MC903 and MC902, and Contilisant, was compared with that induced by standards NXY-059 and QN23 (Table 1). Not surprisingly, and according to the highest cell viability values mentioned above, QNs JMA101A (10–100 µM), JMA98C (10 µM) and MC903 (10–100 µM) exerted the highest neuroprotective effect, being even higher than NXY-059. Again, 10 µM or 100 µM MC903 achieved the neuroprotection values most similar to our ischemic stroke lead-candidate QN23. 

These findings confirm the selection of the QN as a proper scaffold in the search of neuroprotective ligands. Modification of the QN core is not only easily feasible, but also able to provide new functionalities and properties without being detrimental to the OGD-protecting effect. Specially, for the cell viability results after OGD presented above, the preliminary structure–activity relationship (SAR) reported here has afforded no significant deleterious combination of structural moieties for an OGD-protecting new entity, and only four compounds were found neurotoxic at the highest concentration tested. On the other hand, one specific QN, MC903, has been revealed as the best-performing of the set, with an OGD-protecting activity similar to QN23. 

In order to evaluate the suitability of these structures as multipotent hits, assessment of their neuroprotective effect on the specific pathological contributions of AD was required. These results are described below.

### 2.3. Evaluation of Neuroprotection in an Experimental Model of AD in Neuronal Cell Line 

In an independent and almost simultaneous assay, the neuroprotective activity of the target QNs (Figure 1) was carried out at different concentrations (0.3, 1, 3 and 10 μM) against three toxic stimuli related to the neurodegeneration found in AD using melatonin as standard, a well-known neuroprotective agent, for comparative purposes. 

First of all, we used a model of reactive oxygen species generation using a cocktail of mitochondrial blockers, rotenone plus oligomycin A (R/O). As shown in Table 2, QNs JMA98C and MC903 showed an interesting profile against R/O. At the concentrations of 1, 3 and 10 μM, JMA98C produced a significant increase in cell viability against R/O (40.6%, 70.5% and 57.2%, respectively). Similarly, MC903, at 0.3, 1 and 3 μM, showed a potent increase in cell viability (44.1%, 40.4% and 74.9%). The maximum protective effect of both QNs was achieved at the concentration of 3 μM. Next, we analyzed our ligands in a model of tau hyperphosphorylation using okadaic acid (OA), a well-known protein Ser/Thr phosphatase inhibitor. In this model, both QNs showed a significant increase in cell viability against OA at the concentrations of 1 and 3 μM (JMA98C: 52.9% and 78.3%, respectively; MC903: 46.7% and 56.2%, respectively). Finally, and based on the precedent results, we investigated the neuroprotective effect of QNs JMA98C and MC903 following a toxic insult given by A*β*_25–35_, as a largely accepted biological target playing a key role in the progress and development of AD. As shown in Table 2, again QN MC903 showed a significant, potent and dose–response neuroprotective effect of 47.9 ± 8.9% at 3 μΜ, decreasing at 10 μM, and reaching similar lower values, around 28%, at 0.3 μM and 1 μM. Thus, from two different laboratories carrying out independent assays on the same QNs in order to assess their neuroprotective capacities under an experimental ischemia model and against AD-derived toxic insults, QN MC903 was found to provide the best, strong and consistent neuroprotection, identifying it as a potential hit-compound for further preclinical studies.

### 2.4. Virtual Absorption, Distribution, Metabolism, and Excretion (ADME) Properties of Compound MC903

MC903 virtual ADME profile was investigated as a key point for a molecule designed to therapeutically act on the brain. Furthermore, MC903 drug-like properties were predicted using the QikProp program, and violations of the Lipinski’s rule of 5 (ROF) and Jorgensen’s rule of 3 (ROT), estimated. The calculated ADME parameters (Table S1, Supplementary Material) are within the reference ranges, showing no violation for ROF and ROT. This analysis confirms that ligand MC903 possesses the appropriate pharmacokinetic profiles required for distribution in the human body, being permeable to the brain–blood barrier. As such, MC903 has the potential as a lead candidate in AD and stroke drug development.

## 3. Materials and Methods

### 3.1. Synthesis

Target QNs were prepared and purified as shown in the Supplementary Material.

### 3.2. Neuroprotection Analysis in an Ischemia Experimental Model

#### 3.2.1. Primary Neuronal Cultures

Primary neuronal cultures were prepared from cerebral cortex as previously described [16]. Cerebral cortices were obtained from E16 Sprague-Dawley rat embryos, homogenized, and seeded on plastic multidishes precoated with 0.05 mg/mL poly-D-lysine at a density of 2.5 × 10^5^ cells/cm^2^. Cell cultures were kept in a 6.5% CO_2_ atmosphere at 37 °C, in high glucose Dulbecco’s medium supplemented with 15% heat-inactivated (56 °C, 30 min) fetal calf serum. After 24 h, culture medium was replaced by serum-free medium (Dulbecco’s/Ham’s F12, 1:1 vol/vol, 5 mg/mL glucose, 2 mM L-glutamine, and 1 mM sodium pyruvate, and supplemented with 100 μg/mL transferrin, 100 μM putrescine, 20 nM progesterone, 30 nM sodium selenite, and 5 μg/mL insulin). In the reported experiments, 6- to 7-days in vitro (DIV) neuronal cultures were used, which contained 90% β-tubulin isotype III-positive mature neurons, as described previously [17]. All procedures associated with animal experiments were authorized by the Ethics Committee of the Hospital Universitario Ramón y Cajal (Madrid, Spain).

#### 3.2.2. Experimental Ischemia in Neuronal Cultures and Treatments

In order to induce experimental ischemia, primary neuronal cultures were subjected to oxygen–glucose deprivation (OGD) [18]. The 6- to 7-DIV cultures were washed and placed in glucose-free Dulbecco’s medium, which was previously bubbled with 95% N_2_/5% CO_2_ for 30 min, and kept in a humidified anaerobic chamber containing a gas mixture of 95% N_2_/5% CO_2_ at 37 °C for 4 h (OGD4h). Control group was placed in glucose-supplemented Dulbecco’s medium, and kept in the normoxic incubator for 4 h. After 4 h of incubation under anoxic (OGD4h) or normoxic (control) condition, culture cells were quickly placed in serum-free normoglycemic culture medium and reoxygenation condition, then compounds or vehicle were added and cells were maintained in normoxic and normoglycemic conditions to recovery for 24 h. Vehicle experimental group (R24h) included the same amounts of vehicle than the compounds tested (final concentration < 0.5% ethanol). After the recovery for 24 h, cell viability was determined. Each group assayed was assigned a random order to blindly perform the experimental procedure. Compound assays were carried out independently four to eight different times with different batches of cultures, and each experiment was run in quadruplicate.

#### 3.2.3. Cell Viability Assay

For the assessment of the neuroprotective effect of the compounds against OGD, cell viability was evaluated by quantification of living, metabolically active cells, as determined by the photometric reduction of 3-(4,5-dimethylthiazol-2-yl)-2,5-diphenyl tetrazolium bromide (MTT) (Roche) to a blue formazan product. MTT assay was performed after the recovery period—except for the OGD 4 h group, for which MTT was added right after the end of the OGD period, with no recovery—by treating cells with 0.2 mg/mL MTT in the culture medium for 1.5 h at 37 °C in a 6.5% CO_2_ atmosphere. After incubation, cells were lysed with an equal volume of lysis solution (10 mM HCl, 10% SDS) overnight. MTT reduction was quantified by the absorbance measurement at 595 nm (690 nm as reference). Decreased MTT activity denotes an impairment of mitochondrial function and is considered to be indicative of cell damage.

### 3.3. Neuroprotection Analysis in AD Experimental Models

#### 3.3.1. SHSY-5Y Cell Culture

The neuroblastoma SH-SY5Y cell line was obtained from Sigma-Aldrich (Madrid, Spain), maintained and cultured in a 1:1 mixture of Eagle’s minimum essential medium (EMEM) and F12 media supplemented with 10% of fetal bovine serum (FBS), according to procedures previously described [8]. Cultures were seeded into flasks and maintained at 37 °C in a humidified atmosphere of 5% CO_2_ and 95% air. For viability assays, SH-SY5Y cells were seeded in 96-well plates at a density of 50,000 cells/well following the procedure reported [8]. In this study, all cells were used at a low passage number (<14).

#### 3.3.2. Neuroprotection Studies

To study the potential neuroprotective effect of the compounds, cells were pre-incubated with the compounds at 0.3, 1, 3 or 10 μM for 24 h. Then, cells were incubated with 30 μM Rotenone and 10 μM Oligomycin-A (R/O) [19], Okadaic Acid (20 nM) [20] or Amyloid-*β*25-35 (30 μM) (Sigma-Aldrich, Madrid, Spain) with or without compounds at different concentrations for 24 h. At the end of the experiment, cell viability was measured using the MTT method described in Section 3.2.3.

### 3.4. Statistical Analysis

Results obtained for each treatment were averaged and independently analyzed, and the treatment information was kept concealed throughout the study. Data were represented as mean ± SE. Analysis of variance (ANOVA) was performed to compare the data between multiple concentrations, following post hoc test when analysis of variance was significant. Statistical significance level was set at α = 0.05 using Prism statistical software 5.0 (GraphPad).

### 3.5. ADME Analysis

The ADME properties of compound MC903 were evaluated with QikProp module of Schrödinger (QikProp, version 5.1, Schrödinger, LLC, New York, NY, USA, January 2017) [21,22,23]. 

## 4. Conclusions

In this work we disclosed the preliminary results that we obtained in the search for single and novel agents for the potential combined treatment of ischemic stroke and AD. Contrary to other reported approaches [14,15], here we addressed for the first time a rationally-driven drug design using a fragment-based drug design strategy based on the juxtaposition of selected pharmacophores from QN23 and Contilisant, our most advanced lead-compounds for ischemic stroke and AD treatment, respectively. Thus, looking for new nitrones as neuroprotective-antioxidant agents, able not only to decrease ROS levels but also to bind to biological targets and increase the level of neurotransmitters—a key deficit found in the brain of AD patients—we synthesized QNs JMA101A, JMA98C, DDI89, DDI88, JMA12A, JMA11A, MC903, and MC902 (Figure 1) and subjected them to appropriate phenotypic assays for ischemic injury and AD. As a result, we were able to identify MC903 as a hit-agent. Regarding the structure, MC903 bears the same functional groups at the same positions as QN23, except by a *N*-propargylpiperazine substituted at the other nitrogen by a linear three methylene motif linked to O(C6), instead of a methoxy group. Taking into account the results obtained by the preliminary SAR reported here, this functional and pharmacophore arrangement seems critical for the observed activities and should be taken into consideration for further developments. Thus, MC903 is a new and small QN for the potential combined therapy of ischemic stroke and AD, showing strong neuroprotective properties on primary neuronal cultures under OGD conditions as experimental ischemia model, and on neuronal line cells against to R/O, OA or A*β*_25–35_ treatments, which mimic the effects found in AD. Based on these promising results, our efforts are now directed to improve the observed pharmacological profile of hit-MC903 by the design, synthesis and biological evaluation of new related QNs, including the pertinent enzymatic and receptor binding analyses, looking for a lead-QN to submit to suitable ischemic stroke and AD animal models. The present results pave the way for new therapeutic insights and developments in the search for new agents for the combined treatment of ischemic stroke and AD.

## 5. Patents

Marco Contelles, José Luis; Alcázar González, Alberto; Egea Máiquez, Javier; Palomino Antolín, Alejandra. Derivados de quinolilnitronas para su uso en la prevención y el tratamiento de la isquemia cerebral, ictus isquémico y enfermedades neurodegenerativas. EP202130259.

## Figures and Tables

**Table 1:** Neuroprotective activity for the new QNs, standards QN23, NXY-059, and Contilisant in neuronal cultures subjected to OGD conditions. ^a^

QN	Concentration (μM)	Neuroprotection (%)
NXY-059	100	42.11 ± 2.27
	250	51.41 ± 2.13
	500	45.81 ± 1.56
QN23	10	49.16 ± 0.90
	100	78.11 ± 1.18 **
	250	60.80 ± 1.29 **
	500	17.86 ± 0.78
JMA101A	1	35.98 ± 0.64
	10	54.19 ± 1.24
	100	57.23 ± 0.46 *
	250	9.22 ± 0.32
JMA12A	1	10.32 ± 0.27
	10	38.89 ± 1.51
	100	31.79 ± 0.80
	250	<0
JMA98C	0.1	42.81 ± 1.02
	1	46.57 ± 0.60
	10	63.82 ± 0.81 **
	100	<0
MC902	0.1	24.99 ± 0.51
	1	30.34 ± 0.59
	10	45.56 ± 0.54
	100	46.97 ± 0.34
MC903	1	23.40 ± 0.54
	10	72.45 ± 1.64 **
	100	72.67 ± 1.34 **
	250	13.75 ± 0.46
JMA11A	0.1	28.66 ± 0.36
	1	30.12 ± 0.51
	10	44.01 ± 0.71
	100	<0
DDI88	0.1	31.67 ± 0.44
	1	40.91 ± 0.87
	10	39.54 ± 0.54
	100	48.39 ± 1.70
DDI89	1	13.49 ± 0.66
	10	39.63 ± 1.06
	100	44.49 ± 2.25
	250	<0
Contilisant	0.1	5.81 ± 0.10
	1	41.27 ± 1.35
	10	34.62 ± 0.46
	20	47.56 ± 0.36
	50	50.24 ± 1.11
	100	15.22 ± 0.43

^a^ Neuroprotection was defined as the percentage to reach the control value, defined as 100%, from R24h value, defined as 0%. * *p* < 0.05 and ** *p* < 0.01, compared with NXY059 (250 µM), by ANOVA and Dunnett’s post-test. Statistical significances of the data lower than 250 µM NXY059 value were not shown.

**Table 2:** Quantitative data of the protective effect of synthetic QNs and melatonin on SH-SY5Y cell death induced by 30 µM rotenone plus 10 µM oligomycin A (R/O), 20 nM okadaic acid (OA) and 30 µM A*β*_25–35_.

QN (μM)		R/O	OA	A*β*_25–35_
JMA98C	0.3	26.0 ± 24.5	37.5 ± 9.5	14.5 ± 7.9
	1	40.6 ± 1.5 *	52.9 ± 8.8 *	14.2 ± 13.7
	3	70.5 ± 9.7 ***	78.3 ± 16.2 ***	27.8 ± 9.6
	10	57.2 ± 16.3 **	20.8 ± 12.8	3.3 ± 15.2
JMA101A	0.3	5.7 ± 27.3	35.3 ± 14.4	n.d.
	1	26.9 ± 28.3	37.0 ± 21.1	n.d.
	3	22.0 ± 29.2	38.3 ± 4.9	n.d.
	10	24.9 ± 29.1	17.5 ± 23.0	n.d.
JMA11A	0.3	52.8 ± 37.9	21.1 ± 12.8	n.d.
	1	16.7 ± 29.2	29.7 ±10.7	n.d.
	3	37.9 ± 8.8	8.9 ±20.0	n.d.
	10	31.5 ± 30.1	21.5 ± 17.4	n.d.
JMA12A	0.3	11.1 ± 30.0	28.8 ± 26.4	n.d.
	1	39.23± 17.7	20.8 ±13.0	n.d.
	3	11.0± 30.3	n.p.	n.d.
	10	36.0 ± 21.2	22.0 ± 23.2	n.d.
MC902	0.3	10.3 ± 13.3	n.p.	n.d.
	1	32.3 ± 20.3	n.p.	n.d.
	3	n.p.	n.p.	n.d.
	10	n.p.	n.p.	n.d.
MC903	0.3	44.1 ± 9.2 ***	33.3 ± 10.4	29.7 ± 3.6 *
	1	40.4 ± 10.8 ***	46.7 ± 13.4 *	28.9 ± 6.8 *
	3	74.9 ± 5.3 ***	56.2 ± 11.1 **	47.9 ± 8.9 ***
	10	14.4 ± 7.0	n.p.	27.7 ± 8.0 *
DDI88	0.3	n.p.	n.p.	n.d.
	1	n.p.	n.p.	n.d.
	3	n.p.	n.p.	n.d.
	10	n.p.	n.p.	n.d.
DDI89	0.3	n.p.	n.p.	n.d.
	1	n.p.	n.p.	n.d.
	3	n.p.	n.p.	n.d.
	10	n.p.	37.2 ± 27.2	n.d.
Melatonin	10 nM	53.7 ± 10.6 **	53.9 ± 9.5 **	65.3 ± 4.3 **

Data are expressed as % neuroprotection ± SE of triplicate of at least five different cultures. Compounds were assayed at 0.1, 0.3, 1 and 3 μM. *** *p* <0.001, ** *p* < 0.01, * *p* < 0.05, n.p.: not protective, with respect to control. n.d.: not determined.

## Data Availability

Data is contained within the article and Supplementary Material.

## Supplementary Materials

The following are available online at https://www.mdpi.com/article/10.3390/ph14090861/s1, Synthesis of quinolylnitrones (methods, analytical and spectroscopic data), NMR spectra, and prediction of ADME properties for compound MC903, Table S1: Physicochemical properties for compound MC903 calculated using Qikprop.
