# Supplementary material for: Privileged Quinolylnitrones for the Combined Therapy of Ischemic Stroke and Alzheimer’s Disease
Source: Pharmaceuticals (Basel). 2021 Aug 27;14(9):861. doi: 10.3390/ph14090861 (PMC8465398; doi:10.3390/ph14090861)
Supplement: Supplementary file 1 [file pharmaceuticals-14-00861-s001.zip › pharmaceuticals-1350672-supplementary.pdf]

Supplementary Materials

Privileged Quinolylnitrones for the Combined Therapy of Ischemic Stroke and Alzheimer's Disease

José M. Alonso,[1,‡] Alejandro Escobar-Peso,[2,‡] Alejandra Palomino-Antolin,[3,‡] Daniel Diez-Iriepa,[1,4] Mourad Chioua,[1] Emma Martínez-Alonso,[2] Isabel Iriepa,[4,5] Javier Egea,[*,3] Alberto Alcàzar,[*,2] and José Marco-Contelles[*,1]

[1] Laboratory of Medicinal Chemistry (IQOG, CSIC), Juan de la Cierva 3, Madrid 28006, Spain; xosemag@yahoo.es (J.M.A.), daniel.diezi@uah.es (D.D.-I.), mchioua@gmail.com (M.C.), jlmarco@iqog.csic.es (J.M.-C.)
[2] Department of Research, IRYCIS, Hospital Ramón y Cajal, Ctra. Colmenar km 9.1, Madrid 28034, Spain; alejandro.escobar@hrc.es (A.E.-P.), emma.martinez@hrc.es (E.M.-A.), alberto.alcazar@hrc.es (A.A.)
[3] Instituto de Investigación Sanitaria, Servicio de Farmacología Clínica, Hospital Universitario de la Princesa, Diego de León 62, Madrid 28006, Spain; apantolin@gmail.com (A.P-A.); javier.egea@inv.uam.es (J.E)
[4] Departamento de Química Orgánica y Química Inorgánica, Universidad de Alcalá, Ctra. Madrid-Barcelona km. 33.6, Alcalá de Henares 28871, Spain; isabel.iriepa@uah.es (I.I.)
[5] Institute of Chemical Research Andrés M. del Río, Alcalá University, Alcalá de Henares 28805, Spain

* Correspondence: javier.egea@inv.uam.es (J.E.); alberto.alcazar@hrc.es (A.A.); jlmarco@iqog.csic.es (J.M.-C)

CONTENTS

1. Synthesis of quinolylnitrones……………………………………………S2-S12

2. NMR spectra of quinolylnitrones……………………………………..S13-S23

3. Prediction of ADME properties for compound MC903………………..S24-S25

1. Synthesis of quinolylnitrones

1.1. Materials and methods. Melting points were determined on a Köffler apparatus, and are uncorrected. ^{1}H NMR and ^{13}C NMR spectra were recorded in $CDCl_3$ or DMSO-d_6 at 300 MHz and at 75 MHz, respectively, using solvent peaks [$CDCl_3$: 7.26 (D), 77.2 (C) ppm; and DMSO-d_6: 2.49 (D), 39.52 (C) ppm] as internal reference. The assignment of chemical shifts is based on standard NMR experiments (^{1}H, ^{13}C-DEPT, ^{1}H, ^{1}H-COSY, gHSQC, gHMBC). Mass spectra were recorded on a GC/MS spectrometer with an API-ES ionization source. Elemental analyses were performed at CNQO (CSIC, Spain). TLC were performed on silica F254 and detection by UV light at 254 nm or by charring with either ninhydrin, anisaldehyde or phosphomolybdic-H_2SO_4 reagents. Anhydrous solvents were used in all experiments. Column chromatography was performed on silica gel 60 (230 mesh). **General procedure for the synthesis of nitrones (A).** A solution of the corresponding carbaldehyde (1 mmol), Na_2SO_4 (3 mmol), triethylamine (TEA) (2 mmol) and the appropriate *N*-alkylhydroxylamine hydrochloride (1.5 mmol) in THF/EtOH (5 mL, 4:1) was heated at 90 °C for 1-7 h under MW irradiation (MWI). After that time, the solvent was evaporated and the crude mixture was purified on column chromatography using the indicated mixtures of solvents. **General procedure for the synthesis of nitrones (B).** A solution of the corresponding carbaldehyde (1 mmol), Na_2SO_4 (2 mmol), AcONa (1.2 mmol) and the appropriate *N*-alkylhydroxylamine hydrochloride (1.2 mmol) in EtOH (5 mL) was heated at 95 °C for 2-3 h under MWI. Then, the solvent was evaporated and the crude mixture was purified by column chromatography using the indicated mixtures of solvents.

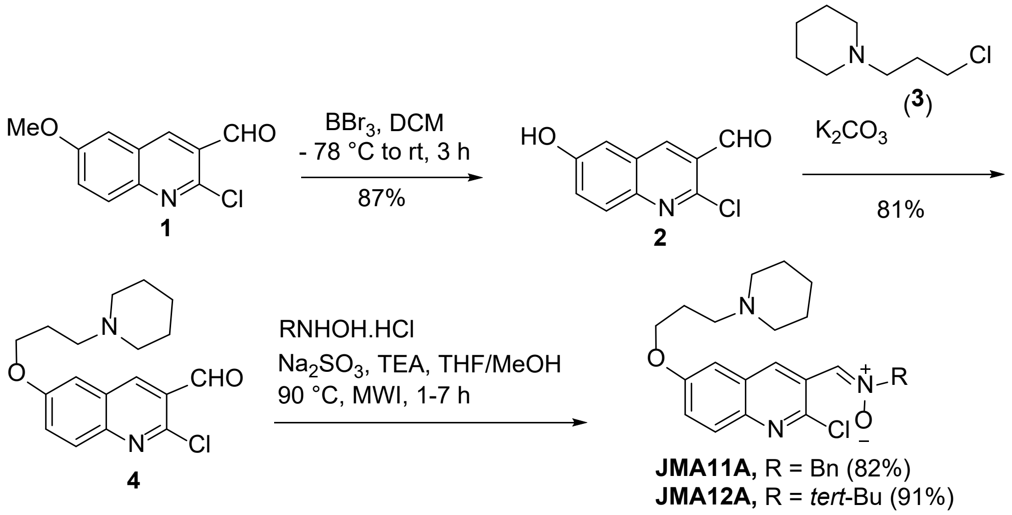

1.2. Synthesis of quinolylnitrones JMA101A, JMA98C, JMA12A, and JMA11A

1.2.1. 2-Chloro-6-hydroxyquinoline-3-carbaldehyde (2). A solution of commercial 2-chloro-6-methoxyquinoline-3-carbaldehyde (**1**) (500 mg, 2.262 mmol) in methylene chloride (DCM) (14 mL), cooled at - 78 °C, was treated with BBr_3 (7,9 mL, 1M in DCM, 3.5 equiv), and left stirring at the same temperature for 30 min. After that time the mixture was left to reach rt, and stirred for additional 3 h. Then, water (5 mL) was carefully added at 0 °C, and the mixture was extracted using AcOEt/H_2O. The organic phases were collected, washed with brine, and dried with $MgSO_4$, filtered and the solvent was evaporated under reduced pressure to yield clean product **2** (405 mg, 87%), showing coincident analytical data to previously reported structure.[1]

1.2.2. 2-Chloro-6-(3-(piperidin-1-yl)propoxy)quinoline-3-carbaldehyde (**4**). A solution of K_2CO_3 (200 mg, 1.449 mmol) in water (0,5 mL) was added to a solution of commercial 1-(3-chloropropyl)piperidine (**3**) (100 mg, 0.483 mmol) in $CHCl_3$ (3 mL). The mixture was vigorously stirred and heated at 80 °C for 3 h, and then left 16 h at 40 °C. After that time, the solvent was evaporated under reduced pressure and the crude mixture was purified on column chromatography (hexane/AcOEt/MeOH 1:1:1) to yield compound **4** as a white solid (130 mg, 81%): mp 79-81°C; IR (KBr) ν 1696 cm^{-1}; ^1H NMR (300 MHz, $CDCl_3$) δ 10.47 (s, 1H), 8.55 (s, 1H), 7.88 (d, *J* = 9.2 Hz, 1H), 7.43 (dd, *J*= 9.2, 2.7 Hz, 1H), 7.13 (d, *J*= 2.7 Hz, 1H), 4.08 (t, *J*= 6.3 Hz, 2H), 2.45-2.43 (m, 6H), 2.10-1.93 (m, 2H), 1.57-1.55 (m, 4H), 1.41-1.39 (m, 2H); ^{13}C NMR (75 MHz, $CDCl_3$) δ 189.8 (CH=O), 158.6 (C, Ar), 148.0 (C, Ar), 146.1 (C, Ar), 139.0 (CH, Ar), 130.2 (CH, Ar), 128.2 (C, Ar), 127.1 (CH, Ar), 126.8 (C, Ar), 107.6 (CH, Ar), 67.4

(CH_2), 56.1 (CH_2), 55.0 (2 CH_2), 26.8 (CH_2), 26.1 (2 CH_2), 24.6 (CH_2); MS (EI) *m/z*: 332.1 (91) [M^+]. HRMS (ESI_ACN). Calcd. for $C_{18}H_{21}ClN_2O_2$: 332.12916. Found: 332.12926.

1.2.3. (Z)-*N*-Benzyl-1-(2-chloro-6-(3-(piperidin-1-yl)propoxy)quinolin-3-yl)methanimine oxide (**JMA11A**). Following the general method **A** for the synthesis of nitrones, a solution of carbaldehyde **4** (110 mg, 0.342 mmol), Na_2SO_4 (129 mg, 1.026 mmol), TEA (0.09 mL, 0.684 mmol) and *N*-benzylhydroxylamine hydrochloride (81 mg, 0.512 mmol) in THF/EtOH (5 mL, 4:1) was heated at 90 °C for 1 h under MWI. After that time, the solvent was evaporated and the crude mixture was purified on column chromatography (MeOH/AcOEt 1:1) to yield compound **JMA11A** as a pale yellow solid (122 mg, 82%): mp 103-4 °C; IR (KBr) ν 1616 cm^{-1}; ^1H NMR (300 MHz, $CDCl_3$) δ 10.16 (s, 1H), 8.08 (s, 1H), 7.83 (d, *J*= 9.2 Hz, 1H), 7.57-7.48 (m, 2H), 7.48-7.39 (m, 3H), 7.35 (dd, *J*= 9.2, 2.7 Hz, 1H), 7.10 (d, *J*= 2.8 Hz, 1H), 5.16 (s, 2H), 4.11 (t, *J*= 6.0 Hz, 2H), 2.78-2.73 (m, 6H), 2.30-2.08 (m, 2H), 1.82-1.77 (m, 4H), 1.55-1.53 (m, 2H); ^{13}C NMR (75 MHz, $CDCl_3$) δ 157.9 (C. Ar), 146.1 (C, Ar), 143.4 (C, Ar), 136.4 (CH=N), 133.1 (C, Ar), 129.9 (2 CH, Ar), 129.76 (3 CH, Ar), 129.72 (C, Ar), 129.5 (2 CH, Ar), 128.4 (C, Ar), 124.6 (CH, Ar), 122.8 (C, Ar), 107.5 (C, Ar), 72.7 (CH_2), 66.7 (CH_2), 56.0 (CH_2), 54.6 (2 CH_2), 25.9 (CH_2), 25.0 (2 CH_2), 23.9 (CH_2); MS (EI): 437.2 (3) [M^+]; 421.2 (100) [M^+-O]; 402.2 (8) [M^+-Cl]. HRMS (ESI_ACN). Calcd for $C_{25}H_{28}ClN_3O_2$: 437.187. Found: 437.18632. Anal. Calcd for $C_{25}H_{28}ClN_3O_2$.2/3 H_2O: C, 66.73; H, 6.57; N, 9.34. Found: C, 66.67; H, 6.28; N, 9.09.

1.2.4. (Z)-*N-tert*-Butyl-1-(2-chloro-6-(3-(piperidin-1-yl)propoxy)quinolin-3-yl)methanimine oxide (**JMA12A**). Following the general method **A** for the synthesis of nitrones, a solution of carbaldehyde **4** (110 mg, 0.342 mmol), Na_2SO_4 (129 mg, 1.026 mmol), TEA (0.09 mL, 0.684 mmol) and *N-tert*-butylhydroxylamine hydrochloride (64 mg, 0.512 mmol) in THF/EtOH (5 mL, 4:1) was heated at 90 °C for 7 h under MWI. After that time, the solvent was evaporated and the crude mixture was purified on column chromatography (MeOH/AcOEt 1:1) to yield compound **JMA12A** as a yellow solid (125 mg, 91%): mp 118-9 °C; IR (KBr) ν 1617 cm^{-1}; ^1H NMR (300 MHz, $CDCl_3$) δ 10.27 (s, 1H), 8.25 (d, *J*= 0.8 Hz, 1H), 7.84 (d, *J*= 9.2 Hz, 1H), 7.34 (ddd, *J*= 9.2, 2.8, 0.8 Hz, 1H), 7.12 (d, *J*= 2.8 Hz, 1H), 4.12 (t, *J*= 5.9 Hz, 2H), 2.84-2.77 (m, 6H), 2.29-2.27 (m, 2H), 1.89-1.86 (m, 4H), 1.66 (s, 9H), 1.63-1.48 (m, 2H); ^{13}C NMR (75 MHz,

$CDCl_3$) δ 157.8 (C. Ar), 146.8 (C, Ar), 143.2 (C, Ar), 136.1 (CH=N), 129.9 (CH, Ar), 128.6 (C, Ar), 125.7 (CH, Ar), 124.2 (CH, Ar), 123.3 (C, Ar), 107.6 (CH, Ar), 72.8 (C), 66.5 (CH_2), 56.0 (CH_2), 54.5 (2 CH_2), 28.8 (3 C, CH_3), 25.6 (CH_2), 24.7 (2 CH_2), 23.7 (CH_2); MS (EI): 403.3 (2) [M^+]; 386 (100) [M^+-O]. HRMS (ESI_ACN). Calcd. for $C_{22}H_{30}ClN_3O_2$: 403.20204. Found: 403.20265.

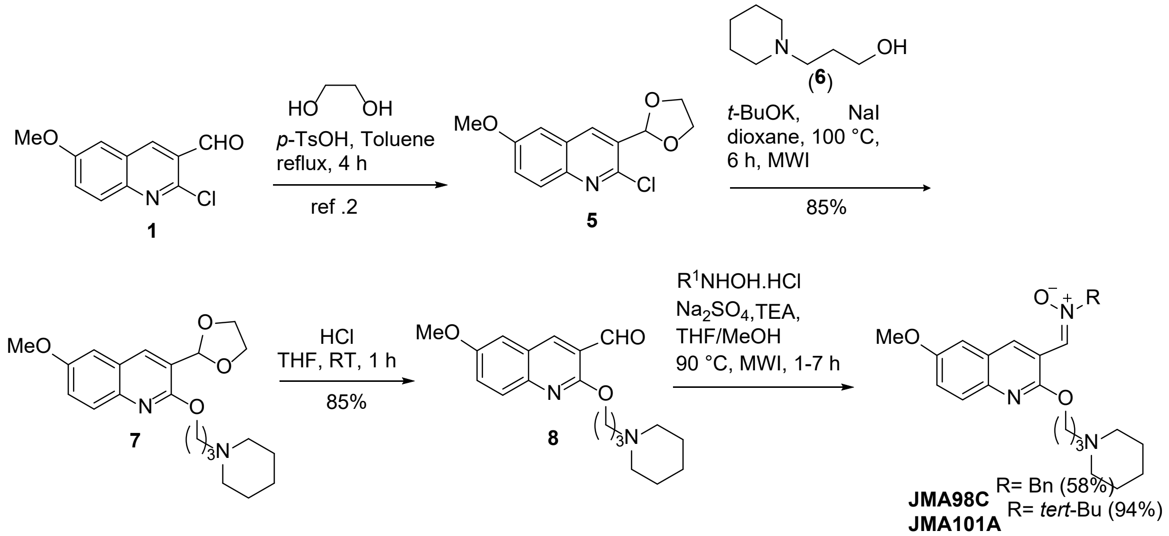

1.2.5. 3-(1,3-Dioxolan-2-yl)-6-methoxy-2-(3-(piperidin-1-yl)propoxy)quinolone (**7**). A solution of 2-chloro-3-(1,3-dioxolan-2-yl)-6-methoxyquinoline (**5**)[2] (200 mg, 0.758 mmol), 3-(piperidin-1-yl)propan-1-ol (**6**) (0.35 mL, 2.272 mmol), *t*-BuOK (170 mg, 1.516 mmol), NaI (114 mg, 0.758 mmol) in dioxane (3 mL) was heated to 110 °C for 6 h under MWI. After that time, the solvent was evaporated and the crude mixture purified on column chromatography (DCM/MeOH 9:1) to yield pure product **7** (257 mg, 85%) as a yellow solid: mp 97-9 °C; ^{1}H NMR (300 MHz, $CDCl_3$) δ 8.05 (s, 1H), 7.65 (d, *J*= 9.1 Hz, 1H), 7.25-7.16 (m, 1H), 7.00 (d, *J*= 2.8 Hz, 1H), 6.07 (s, 1H), 4.48 (t, *J*= 6.3 Hz, 2H), 4.16-3.95 (m, 4H), 3.82 (s, 3H), 2.52 (m, 6H), 2.06 (m, 2H), 1.60 (m, 4H), 1.41 (m, 2H); ^{13}C NMR (75 MHz, $CDCl_3$) δ 158.8 (C, Ar), 156.5 (C, Ar), 142.5 (C, Ar), 135.3 (CH, Ar), 128.6 (CH, Ar), 125.7 (C, Ar), 122.5 (C, Ar), 121.9 (CH, Ar), 106.9 (CH, Ar), 99.9 (CH), 65.7 (3 CH_2), 64.7 (CH_2), 56.4 (CH_2), 55.9 (CH_3), 54.8 (2 CH_2), 26.4 (CH_2), 25.7 (CH_2), 24.4 (CH_2); MS (EI): 372.1 (2) [M^+]. HRMS (ESI_ACN). Calcd. for $C_{21}H_{28}N_2O_4$: 372.20491. Found: 372.20556.

1.2.6. 6-Methoxy-2-(3-(piperidin-1-yl)propoxy)quinoline-3-carbaldehyde (**8**). HCl (0.75 mL, 2M) was added dropwise over a solution of compound **7** (100 mg, 0.25

mmol) in THF (4 mL) at rt. After 1 h, the mixture was diluted with DCM and extracted with $NaHCO_3$ (3x5 mL), brine, and dried over $MgSO_4$. After filtration and evaporation of the solvent, pure product **8** (70 mg, 85%) was obtained as a thick yellow gum: IR (KBr) ν 2933, 1689, 1603 cm^{-1}; ^1H NMR (300 MHz, $CDCl_3$) δ 10.34 (s, 1H), 8.39 (s, 1H), 7.65 (d, *J*= 9.2 Hz, 1H), 7.32 (dd, *J*= 9.2, 2.8 Hz, 1H), 7.03 (d, *J*= 2.8 Hz, 1H), 4.60 (t, *J*= 5.8 Hz, 2H), 3.83 (s, 3H), 3.57-3.06 (m, 6H), 2.51-2.23 (m, 2H), 1.85-1.82 (m, 4H), 1.41 (m, 2H); ^{13}C NMR (75 MHz, $CDCl_3$) δ 189.4 (C=O), 159.1 (1C, Ar), 157.2 (1C, Ar), 144.8 (1C, Ar), 140.1 (1CH, Ar), 129.0 (1CH, Ar), 125.6 (1CH, Ar), 125.5 (1C, Ar), 120.0 (1C, Ar), 107.5 (1CH, Ar), 63.7 (CH_2), 56.0 (Me), 55.7 (CH_2), 53.8 ($2CH_2$), 24.0 (CH_2), 23.0 ($2CH_2$), 22.5 (CH_2); MS (EI): 328.1 (12) [M^+]. HRMS (ESI_ACN). Calcd. for $C_{19}H_{24}N_2O_3$: 328.17869. Found: 328.17938.

1.2.7. (*Z*)-*N*-Benzyl-1-(6-methoxy-2-(3-(piperidin-1-yl)propoxy)quinolin-3-yl)methanimine oxide (**JMA98C**). Following general procedure **A** for the synthesis of nitrones, starting from aldehyde **8** (70 mg, 0.213 mmol), *N*-benzylhydroxylamine hydrochloride (50 mg, 0.321 mmol), Na_2SO_4 (154 mg, 0.639 mmol) and TEA (59 μL, 0.426 mmol) in THF (1.5 mL), and after purification on column chromatography (DCM/MeOH 15:1), expected nitrone **JMA98C** was obtained as an off white solid (53 mg, 58%): mp 183-4 °C; IR (KBr) ν 1596 cm^{-1}; ^1H NMR (300 MHz, $CDCl_3$) δ 9.95 (s, 1H), 7.99 (s, 1H), 7.57 (d, *J*= 9.0 Hz, 1H), 7.49-7.47 (m, 2H), 7.36-7.33 (m, 2H), 7.20-7.17 (m, 2H), 7.01 (br s, 1H), 5.10 (s, 2H), 4.43 (t, *J*= 6.2 Hz, 2H), 3.79 (s, 3H), 2.56-2.50 (m, 6H), 2.08-2.02 (m, 2H), 1.67-1.63 (m, 4H), 1.46-1.44 (m, 2H); ^{13}C NMR (75 MHz, $CDCl_3$) δ 156.7 (C, Ar), 142.1 (C, Ar), 136.6 (CH), 133.8 (C, Ar), 129.7 (3 CH, Ar), 129.3 (CH, Ar), 129.3 (2 CH, Ar), 128.9 (CH, Ar), 128.4 (CH, Ar), 126.0 (C, Ar), 122.8 (CH, Ar), 115.6 (C, Ar), 107.7 (CH, Ar), 72.0 (CH_2), 64.8 (CH_2), 56.3 (CH_2), 55.9 (CH_3), 54.8 (2 CH_2), 26.4 (CH_2), 25.7 (2 CH_2), 24.3 (CH_2); MS (EI): 433.1 (9) [M^+], 416.1 (29) [M^+-O]. HRMS (ESI_ACN). Calcd. for $C_{26}H_{31}N_3O_3$ 433.23654. Found 433.23754. Anal. Calcd for $C_{26}H_{31}N_3O_3.2H_2O$: C, 66.50; H, 7.51; N, 8.95. Found: C, 66.59; H, 7.29; N, 9.08.

1.2.8. (*Z*)-*N-tert*-Butyl-1-(6-methoxy-2-(3-(piperidin-1-yl)propoxy)quinolin-3-yl)methanimine oxide (**JMA101A**). Following general procedure procedure **A** for the synthesis of nitrones, starting from aldehyde **8** (70 mg, 0.213 mmol), *N-tert*-butylhydroxylamine hydrochloride (40 mg, 0.321 mmol), Na_2SO_4 (154 mg, 0.639

mmol) and TEA (59 μL, 0.426 mmol) in THF (1.5 mL), and after purification on column chromatography (DCM/MeOH, 20:1), expected nitrone **JMA101A** was obtained as a yellow solid (79 mg, 94%): mp 150-2 °C; IR (KBr) ν 1617 cm^{-1}; ^{1}H NMR (300 MHz, $CDCl_3$) δ 10.05 (s, 1H), 8.06 (s, 1H), 7.59 (d, *J*= 9.0 Hz, 1H), 7.23-7.12 (m, 1H), 7.04 (d, *J*= 2.8 Hz, 1H), 4.46 (t, *J*= 6.4 Hz, 2H), 3.80 (s, 3H), 2.56-2.32 (m, 6H), 2.07-1.97 (m, 2H), 1.60-1.57 (m, 4H), 1.58 (s, 9H), 1.47-1.33 (m, 2H); ^{13}C NMR (75 MHz, $CDCl_3$) δ 156.7 (C, Ar), 142.0 (C, Ar), 136.2 (CH), 128.3 (CH, Ar), 126.1 (C, Ar), 124.5 (CH, Ar), 122.5 (CH, Ar), 116.0 (C, Ar), 107.8 (CH, Ar), 71.8 (C), 65.1 (CH_2), 56.8 (CH_2), 55.8 (CH_3), 55.1 (2 CH_2), 28.8 (3 C, CH_3), 28.7 (CH_2), 26.8 (2 CH_2), 26.2 (CH_2), 24.7 (CH_2); MS (EI): 433.1 (9) [M^+], 416.1 (29) [M^+-O]. HRMS (ESI_ACN). Calcd. for $C_{23}H_{33}N_3O_3$: 399.25219. Found: 399.25197. Anal. Calcd for $C_{23}H_{33}N_3O_3$.2/3 H_2O: C, 64.76; H, 8,51; N, 9.85. Found: C, 64.90; H, 8.30; N, 9.83.

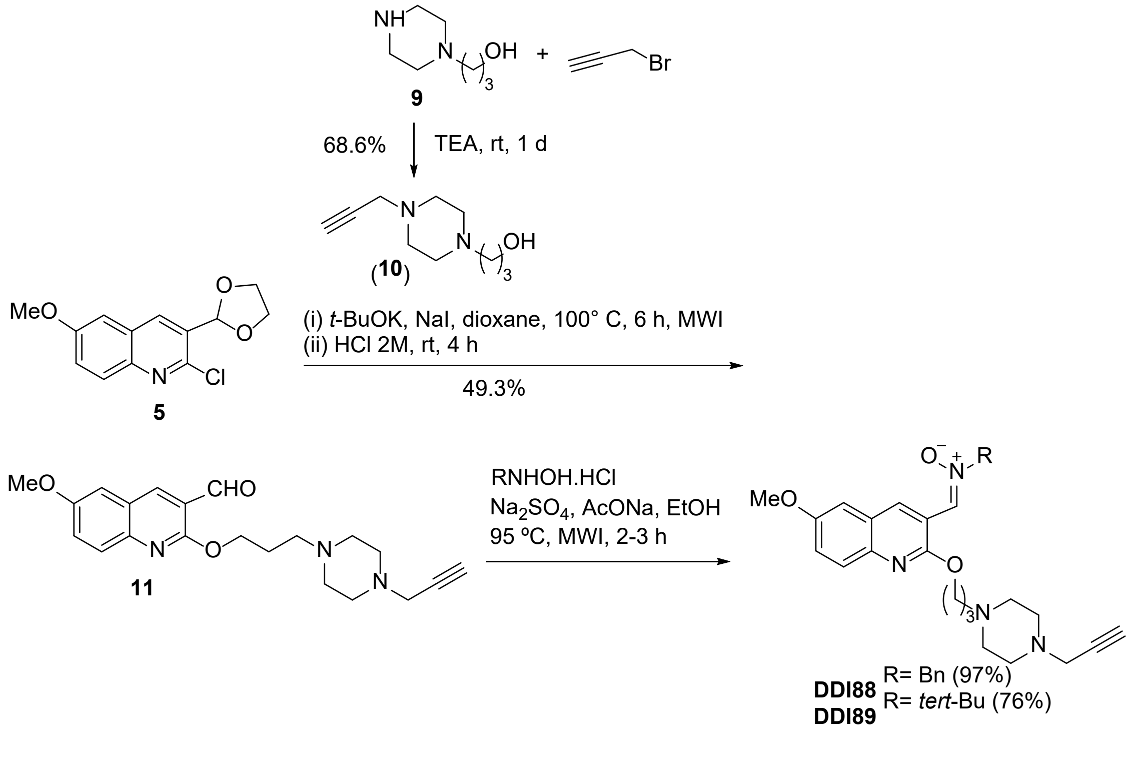

1.3. Synthesis of quinolylnitrones DDI88 and DDI89

1.3.1. 3-(4-(Prop-2-yn-1-yl)piperazin-1-yl)propan-1-ol (10). A mixture of commercial 3-(piperazin-1-yl)propan-1-ol (**9**) (576.8 mg, 4 mmol), TEA (0.557 mL, 4 mmol) and

propargyl bromide (0.490 mL, 4,4 mmol, 80% solution in toluene) in dichloromethane (10 mL) was stirred 1 d at rt. After that time, the crude was diluted with DCM and extracted with $NaHCO_3$ and brine. The organic phase was dried, filtered and evaporated to afford a crude mixture that was purified by column chromatography (DCM/MeOH, 7%) to yield pure product **10** as a pale yellow solid (500.5 mg, 68.6%): mp 56-8 °C; IR (KBr) ν 3152, 2097, 1448, 1147, 721 cm^{-1}; ^1H NMR (300 MHz, $CDCl_3$) δ 3.82-3.77 (m, 2H), 3.29 (d, *J*= 2.5 Hz, 2H, C*H2*C≡CH), 2.77-2.44 (m, 10H), 2.25 (t, *J*= 2.5 Hz, 1H, CH_2C≡C*H*), 1.76-1.66 (m, 2H). HRMS (ESI_ACN). Calcd. for $C_{10}H_{18}N_2O$: 182.1421. Found: 182.1419.

1.3.2. 6-Methoxy-2-(3-(4-(prop-2-yn-1-yl)piperazin-1-yl)propoxy)quinoline-3-carbaldehyde (11). A suspension of compound **5** (90.3 mg, 0.34 mmol), 3-(4-(prop-2-yn-1-yl)piperazin-1-yl)propan-1-ol (**10**) (93.1 mg, 0.51 mmol), *t*-BuOK (76.3 mg, 0.68 mmol), NaI (50.97 mg, 0.34 mmol) in dioxane (0.8 mL) was heated at 110 °C for 6 h under MWI. After that time, the solvent was evaporated and the crude was dissolved in THF (4 mL) and HCl 2M (0.75 mL) was carefully added. The mixture was stirred for 3 h at rt. Then, the mixture was diluted with DCM (10 mL) and extracted with $NaHCO_3$ (aq. saturated solution, 3x10 mL). The organic phase was washed with brine and dried over $MgSO_4$. After filtration, and evaporation of the solvent, the crude was purified by column chromatography (DCM/MeOH, 4%) to yield pure product **11** (69 mg, 49.3%) was isolated as an amorphous solid: ^1H NMR (300 MHz, $CDCl_3$) δ 10.41 (s, 1H), 8.42 (s, 1H), 7.67 (d, *J*= 9.1 Hz, 1H), 7.31 (dd, *J*= 9.2, 2.8 Hz, 1H), 7.05 (d, *J*= 2.8 Hz, 1H), 4.54 (t, *J*= 6.4 Hz, 2H), 3.84 (s, 3H), 3.24 (d, *J*= 2.5 Hz, 2H, C*H2*C≡CH), 2.54 (m, 10H), 2.18 (t, *J*= 2.5 Hz, 1H, CH_2C≡C*H*), 2.11-1.91 (m, 2H); ^{13}C NMR (75 MHz, $CDCl_3$) δ 189.9 (C=O), 160.3 (C, Ar), 157.0 (C, Ar), 145.2 (C, Ar), 138.7 (CH, Ar), 128.9 (CH, Ar), 125.3 (C, Ar), 125.2 (CH, Ar), 120.3 (C, Ar), 107.6 (CH, Ar), 79.2 (CH_2*C*≡CH), 73.6 (CH_2C≡*C*H), 65.1 (CH_2), 56.0 (CH_2), 55.7 (CH_3), 53.5 (2 CH_2), 52.2 (2 CH_2), 47.2 (*C*H_2C≡CH), 26.8 (CH_2); MS (EI): 367.1 (100) [M^+]. HRMS (ESI_ACN). Calcd. for $C_{21}H_{25}N_3O_3$: 367.18959. Found: 367.18950.

1.3.3. (*Z*)-*N*-Benzyl-1-(6-methoxy-2-(3-(4-(prop-2-yn-1-yl)piperazin-1-yl)propoxy)quinolin-3-yl)methanimine oxide (DDI88). Following general procedure **B** for the synthesis of nitrones, starting from aldehyde **11** (100 mg, 0.27 mmol), *N*-

benzylhydroxylamine hydrochloride (52.7 mg, 0.33 mmol), Na_2SO_4 (76.7 mg, 0.54 mmol) and AcONa (27.1 mg, 0.33 mmol) in EtOH (5 mL), and after purification on column chromatography (DCM/MeOH 2%), expected nitrone **DDI88** was obtained as a white solid (125 mg, 97%): mp 130-2 °C; IR (KBr) ν 3303, 2822, 1592, 1347, 1232, 831 cm^{-1}; ^{1}H NMR (400 MHz, $CDCl_3$) δ 9.95 (s, 1H, H-4), 7.90 (s, 1H, H-N=CH), 7.57 (d, *J*= 9.1 Hz, 1H, H-8), 7.45 (dd, *J*= 7.5, 1.9 Hz, 2H, Ph), 7.41-7.29 (m, 3H, Ph), 7.25-7.17 (m, 1H, H-7), 7.02 (d, *J*= 2.8 Hz, 1H, H-5), 5.06 (s, 2H, CH_2Ph), 4.43 (t, *J*= 6.4 Hz, 2H, H-1'), 3.79 (s, 3H, MeO), 3.25 (d, *J*= 2.5 Hz, 2H, $CH_2C\equiv CH$), 2.64-2.42 (m, 10H, H-piperazine, H-3'), 2.19 (t, *J*= 2.4 Hz, 1H, $CH_2C\equiv CH$), 1.95 (m, 2H, H-2'); ^{13}C NMR (101 MHz, $CDCl_3$) δ 155.9 (C, C-2), 155.3 (C, C-4a), 140.8 (C, C-6), 135.1 (CH, C-4), 132.3 (C, C-Ph), 128.2 (CH, C-8), 128.0 (CH, C-Ph), 127.9 (2 CH, C-Ph), 127.4 (CH, N=CH), 126.9 (2 CH, C-Ph), 124.5 (C, C-8a), 121.5 (CH, C-7), 114.1 (C, C-3), 106.2 (CH, C-5), 77.7 (C, $CH_2C\equiv CH$), 72.2 (CH, $CH_2C\equiv CH$), 70.6 (CH_2, CH_2-Ph), 63.5 (CH_2, C-1'), 54.5 (CH_3, MeO), 54.2 (CH_2, C-3'), 52.0 (2 CH_2, C-piperazine), 50.7 (2 CH_2, C-piperazine), 45.8 (CH_2, $CH_2C\equiv CH$), 25.3 (CH_2, C-2'). HRMS (ESI_ACN). Calcd. for $C_{28}H_{32}N_4O_3$: 472,24744. Found: 472.24725. Anal. Calcd.: C, 71.16; H, 6.83; N, 11.86. Found: C, 71.12; H, 6.78; N, 11.83.

1.3.4. (*Z*)-*N*-*tert*-Butyl-1-(6-methoxy-2-(3-(4-(prop-2-yn-1-yl)piperazin-1-yl)propoxy)quinolin-3-yl)methanimine oxide (DDI89). Following general procedure **B** for the synthesis of nitrones, starting from compound **11** (64.1 mg, 0.17 mmol), *N*-*tert*-butylhydroxylamine hydrochloride (26.4 mg, 0.21 mmol), Na_2SO_4 (49.4 mg, 0.35 mmol) and AcONa (17.2 mg, 0.21 mmol) in EtOH (3.2 mL), and after purification by column chromatography (DCM/MeOH 20:1), nitrone **DDI89** was obtained as a cream solid (58.2 mg, 76%): mp 136-8 °C; IR (KBr) ν 3282, 2957, 2814, 1595, 1503, 1343, 1234 cm_{-1}; ^{1}H NMR (400 MHz, $CDCl_3$) δ 10.05 (s, 1H, H-4), 8.05 (s, 1H, N=CH), 7.58 (d, *J*= 9.0 Hz, 1H, H-8), 7.23-7-19 (m, 1H, H-7), 7.04 (d, *J*= 2.9 Hz, 1H, H-5), 4.48 (t, *J*= 6.4 Hz, 2H, H-1'), 3.80 (s, 3H, MeO), 3.25 (d, *J*= 2.4 Hz, 2H, $CH_2C\equiv CH$), 2.64-2.47 (m, 10H, H-piperazine, 3'), 2.19 (t, *J*= 2.4 Hz, 1H, $CH_2C\equiv CH$), 2.08-1.95 (m, 2H, H-2'), 1.57 s, 9H, $C(CH_3)_3$]; ^{13}C NMR (101 MHz, $CDCl_3$) δ 156.2 (C, C-2), 155.3 (C, C-4a), 140.6 (C, C-6), 134.8 (CH, C-4), 126.9 (CH, C-8), 124.7 (C, C-8a), 123.0 (CH, C-N=CH), 121.1 (CH, C-7), 114.6 (C, C-3), 106.4 (CH, C-5), 77.7 (C, $CH_2C\equiv CH$), 72.29 (CH, $CH_2C\equiv CH$), 70.4 [C, $C(CH_3)_3$], 63.4 (CH, C-1'), 54.5

(CH3, MeO), 54.4 (CH, C-3'), 52.1 (2 CH2, C-piperazine), 50.7 (2 CH2, C-piperazine), 45.8 (CH2, *C*H2C≡CH), 27.3 [3 CH3, C(*C*H3)3], 25.4 (CH2, C-2'). HRMS (ESI_ACN). Calcd. for $C_{25}H_{34}N_4O_3$: 438.2615. Found: 438.26309. Anal. Calcd. for $C_{25}H_{34}N_4O_3$: C, 68.47; H, 7.81; N, 12.78. Found: C, 68.39; H, 7.76; N, 12.70.

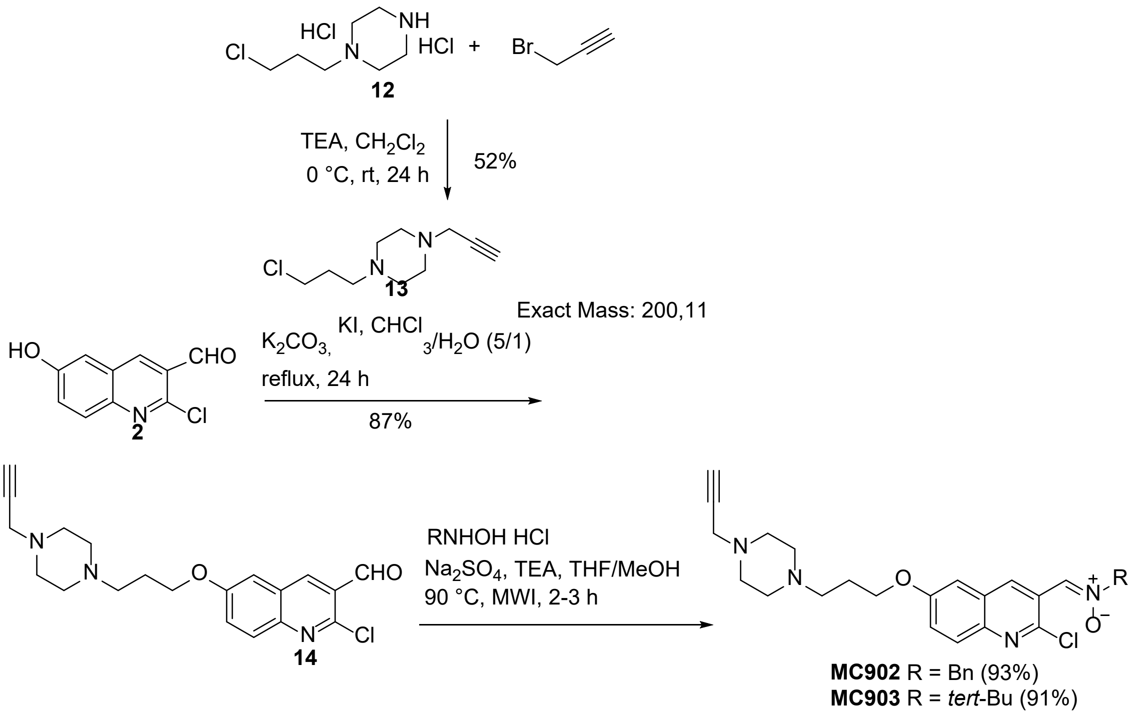

1.4. Synthesis of quinolylnitrones MC903, and MC902

1.4.1. 1-(3-Chloropropyl)-4-(prop-2-yn-1-yl)piperazine (13). To a solution of commercial 1-(3-chloropropyl)piperazine dihydrochloride (**12**) (702 mg, 3 mmol, 1 equiv), TEA (0,84 mL, 6 mmol, 2 equiv) in dry CH_2Cl_2 (5 mL), cooled at 0 °C, propargyl bromide (0,81 mL, 9 mmol, 3 equiv) was added over 30 min, under argon. Then, the mixture was stirred at rt for 24 h, treated with an aq. saturated solution of $NaHCO_3$ (10 mL), the organic layer was separated, washed with brine, dried over anhydrous sodium sulfate, filtered and the solvent was removed. The crude was purified by column chromatography (CH_2Cl_2/MeOH 1%-2%) to give compound **13** (312 mg, 52%) as a white solid: mp > 230 ºC; IR (KBr) ν 3330, 3219, 1612, 1503, 1223 cm^{-1}; ^1H NMR (400 MHz, $CDCl_3$) δ 3.53 (t, *J*= 6.6 Hz, 2H, ClCH2), 3.23 (d, *J*= 2.5 Hz, 2H, HC≡CC*H2*), 2.47-2.39 (m, 10H), 2.18 (t, *J*= 2.5 Hz, 1H, *H*C≡CCH2), 1.88 (p, *J* = 6.7 Hz, 2H, NCH2C*H2*CH2Cl); ^{13}C NMR (101 MHz, $CDCl_3$) δ 78.8 (CH2*C*≡CH),

73.2 ($CH_2C{\equiv}CH$), 56.4 ($ClCH_2$), 55.4 ($NCH_2CH_2CH_2Cl$), 53.1, 51.9 (4 CH_2, piperazine), 46.8 ($CH_2C{\equiv}CH$), 29.9 ($NCH_2CH_2CH_2Cl$). HRMS (ESI_ACN). Calcd. for $C_{10}H_{17}ClN_2$: 200,1080. Found: 200,1080.

1.4.2. 2-Chloro-6-(3-(4-(prop-2-yn-1-yl)piperazin-1-yl)propoxy)quinoline-3-carbaldehyde (14). A solution of compound **13** (300 mg, 1,5 mmol, 1,5 equiv), quimoline **2** (207 mg, 1 mmol, 1 equiv), K_2CO_3 (414 mg, 3 mmol, 3 equiv) and a catalytic amount of KI (17 mg, 0,1 mmol, 0,1 equiv) in $CHCl_3/H_2O$ (5/1, 10 mL) was stirred vigorously for 24 h at 85 °C, and then cooled at rt. The solvents were evaporated, and the residue was purified by flash chromatography (CH_2Cl_2/MeOH 1%-5%) to produce compound **14** (323 mg, 87%) as a pale yellow solid: mp > 230 ºC; IR (KBr) ν 3104, 1570, 1569, 1337 cm^{-1}; ^1H NMR (400 MHz, $CDCl_3$) δ 10.48 (s, 1H, CHO), 8.56 (s, 1H, H-4), 7.89 (d, *J*= 9.2 Hz, 1H, H-8), 7.44 (dd, *J*= 9.2, 2.8 Hz, 1H, H-7), 7.14 (d, *J*= 2.8 Hz, 1H, H-5), 4.09 (t, *J*= 6.3 Hz, 2H, OCH_2), 3.25 (d, *J*= 2.5 Hz, 2H, $HC{\equiv}CCH_2$), 2.56-2.53 (m, 10H), 2.19 (t, *J*= 2.5 Hz, 1H, $HC{\equiv}CCH_2$), 2.00 (p, *J*= 6.6 Hz, 2H, $NCH_2CH_2CH_2O$); ^{13}C NMR (101 MHz, $CDCl_3$) δ 189.5 (C, CHO), 158.2 (C, C-6), 147.6 (C, C-2), 145.8 (C, C-8a), 138.6 (C, C-4), 129.9 (C, C-8), 127.8 (C, C-4a), 126.8 (C, C-7), 126.4 (C, C-3), 107.2 (C, C-5), 78.7 ($CH_2C{\equiv}CH$), 73.3 ($CH_2C{\equiv}CH$), 66.8 (OCH_2), 54.9 (NCH_2), 53.1, 51.8 (4 CH_2, piperazine), 46.8 ($CH_2C{\equiv}CH$), 26.5 ($NCH_2CH_2CH_2O$); MS (EI) *m/z*: 371 [$M^{+\cdot}$, 90%], 332 (20%). HRMS (ESI_ACN). Calcd. For $C_{20}H_{22}ClN_3O_2$: 371,8650. Found: 371,8650.

1.4.3. (*Z*)-*N*-Benzyl-1-(2-chloro-6-(3-(4-(prop-2-yn-1-yl)piperazin-1-yl)propoxy)quinolin-3-yl)methanimine oxide (MC902). Following the general procedure **B** for the synthesis of nitrones, reaction of 2-chloro-6-(3-(4-(prop-2-yn-1-yl)piperazin-1-yl)propoxy)quinoline-3-carbaldehyde (**14**) (93 mg, 0,25 mmol), Na_2SO_4 (71 mg, 2 mmol), AcONa (33 mg, 2 mmol), and *N*-benzylhydroxylamine hydrochloride (48 mg, 1.2 mmol) in EtOH (7 mL), after 1 h, and column chromatography (CH_2Cl_2/MeOH, 1%-8%), gave quinolylnitrone **MC902** (110 mg, 93%) as a white solid: mp > 230 ºC; IR (KBr) ν 3365, 3173, 1588, 1559, 1332 cm^{-1}; ^1H NMR (500 MHz, $CDCl_3$) δ 10.18 (s, 1H, H-4), 8.09 (s, 1H, HC=N), 7.84 (dd, *J*= 9.2, 0.8 Hz, 1H, H-8), 7.56-7.50 (m, 2H, Ph), 7.49-7.39 (m, 3H, Ph), 7.37 (dd, *J*= 9.2, 2.8 Hz, 1H, H-7), 7.12 (d, *J*= 2.8 Hz, 1H, H-5), 5.16 (s, 2H, $PhCH_2$), 4.11 (t, *J*= 6.3 Hz, 2H, OCH_2), 3.31 (d, *J*= 2.5 Hz, 2H, $HC{\equiv}CCH_2$), 2.66-2.57 (m, 10H), 2.26 (t, *J*= 2.5 Hz, 1H,

*H*C≡CCH$_2$), 2.09-2.06 (m, 2H, NCH$_2$C*H$_2$*CH$_2$O); ^{13}C NMR (126 MHz, CDCl$_3$) δ 157.8 (C, C-6), 145.6 (C, C-2), 143.0 (C, C-8a), 136.0 (CH, C-4), 132.7 (C, Ph), 129.5 (CH, CHN), 129.4 (CH, C-8), 129.34 (2 CH, Ph), 129.30 (CH, Ph), 129.1 (2 CH, Ph), 128.1 (C, C-4a), 124.5 (CH, C-7), 122.4 (C, C-3), 107.0 (CH, C-5), 78.7 (CH$_2$*C*≡CH), 73.3 (*C*H$_2$C≡*C*H), 72.3 (PhCH$_2$), 66.5 (OCH$_2$), 55.0, 53.0 (4 CH$_2$-piperazine), 51.7 (N*C*H$_2$CH$_2$CH$_2$O), 46.8 (*C*H$_2$C≡CH), 26.4 (NCH$_2$*C*H$_2$CH$_2$O). HRMS (ESI_ACN). Calcd. for $C_{27}H_{29}ClN_4O_2$: 476.1979 Found: 476.19758. Anal. Calcd. for $C_{27}H_{29}ClN_4O_2$: 67.99; H, 6.13; N, 11.75. Found: C, 68.02; H, 6.20; N, 11.77.

1.4.4. (*Z*)-*N-tert*-Butyl-1-(2-chloro-6-(3-(4-(prop-2-yn-1-yl)piperazin-1-yl)propoxy)quinolin-3-yl)methanimine oxide (MC903). Following the general procedure **B** for the synthesis of nitrones, reaction of 2-chloro-6-(3-(4-(prop-2-yn-1-yl)piperazin-1-yl)propoxy)quinoline-3-carbaldehyde (**14**) (93 mg, 0,25 mmol), Na_2SO_4 (71 mg, 2 mmol), AcONa (33 mg, 2 mmol), and *N-tert*-butylhydroxylamine hydrochloride (50 mg, 1.6 mmol) in EtOH (7 mL), after 2 h, and column chromatography (CH_2Cl_2/MeOH, 1%-7%), gave nitrone **MC903** (100 mg, 91%) as a white solid: mp > 230 ºC; IR (KBr) ν 3365, 3173, 1588, 1559, 1332 cm^{-1}; ^{1}H NMR (300 MHz, CDCl$_3$) δ 10.29 (s, 1H, H-4), 8.27 (s, 1H, HC=N), 7.85 (d, *J*= 9.2 Hz, 1H, H-8), 7.37 (dd, *J* = 9.2, 2.8 Hz, 1H, H-7), 7.15 (d, *J*= 2.8 Hz, 1H, H-5), 4.11 (t, *J*= 6.3 Hz, 2H, OCH$_2$), 3.31 (d, *J*= 2.4 Hz, 2H, C*H$_2$*C≡CH), 2.83-2.42 (m, 10H), 2.26 (t, *J*= 2.4 Hz, 1H, CH$_2$C≡C*H*), 2.07-2.03 (m, 2H), 1.68 [s, 9H, C(CH$_3$)$_3$]; ^{13}C NMR (126 MHz, CDCl$_3$) δ 157.8 (C, C-6), 146.2 (C, C-2), 142.8 (C, C-8a), 135.8 (CH, C-4), 129.4 (CH, C-8), 128.3 (C, C-4a), 125.4 (C, HC=N), 124.2 (CH, C-7), 122.8 (C, C-3), 107.1 (CH, C-5), 78.7 (CH$_2$*C*≡CH), 73.3 (CH$_2$C≡*C*H), 72.4 [*C*(CH$_3$)$_3$], 66.5 (OCH$_2$), 54.9, 53.0 (4CH$_2$, piperazine), 51.7 (N*C*H$_2$CH$_2$CH$_2$O), 46.8 (*C*H$_2$C≡CH), 28.3 [C(*C*H$_3$)$_3$], 26.4 (NCH$_2$*C*H$_2$CH$_2$O). HRMS (ESI_ACN). Calcd. for $C_{24}H_{31}ClN_4O_2$: 442.2136 Found: 442.21333. Anal. Calcd. for $C_{24}H_{31}ClN_4O_2$: 65.07; H, 7.05; N, 12.65. Found: C, 65.13; H, 6.92; N, 12.54.

References

(1) Patel, A. B.; Premlata, K.; Kishor, C. *Catal. Lett.* **2014**, *144*, 1332–1338.
(2) Rajaev, P. V.; Rajaendran, S. P. *Synth. Commun.* **2010**, *40*, 2837–2843.

2. NMR spectra of quinolylnitrones

JMA11A

^{1}H NMR

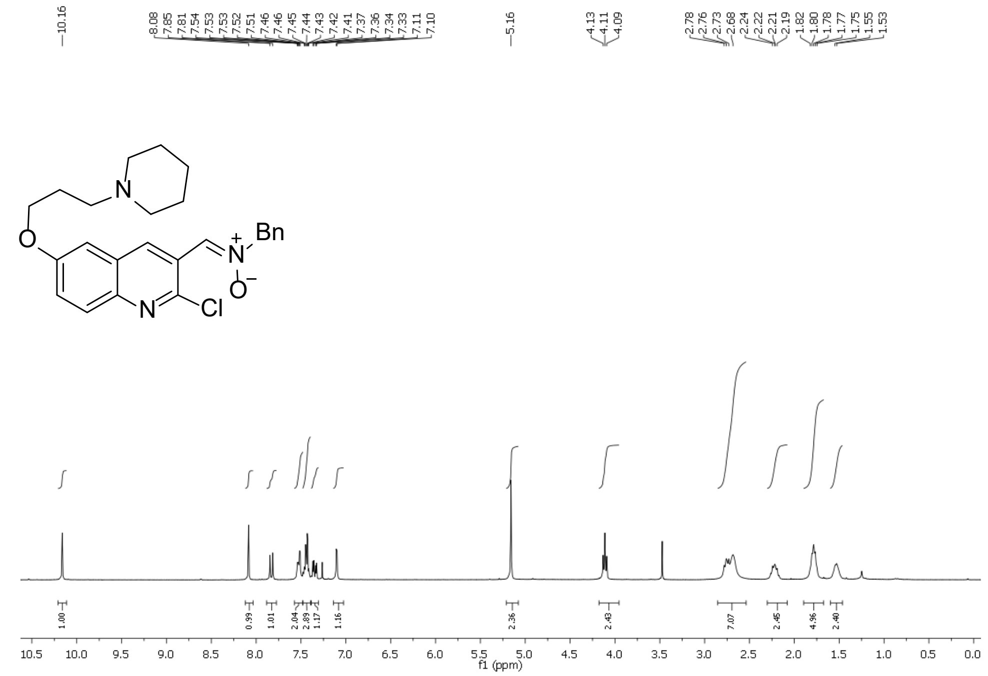

^{13}C NMR

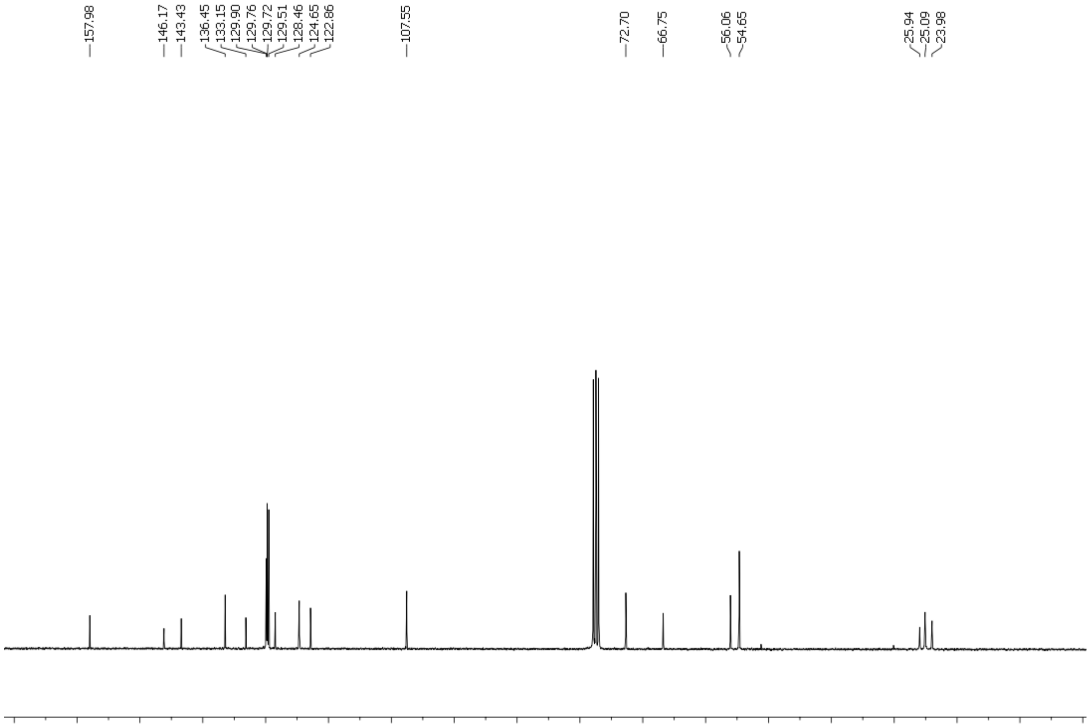

^{13}C NMR vs DEPT NMR

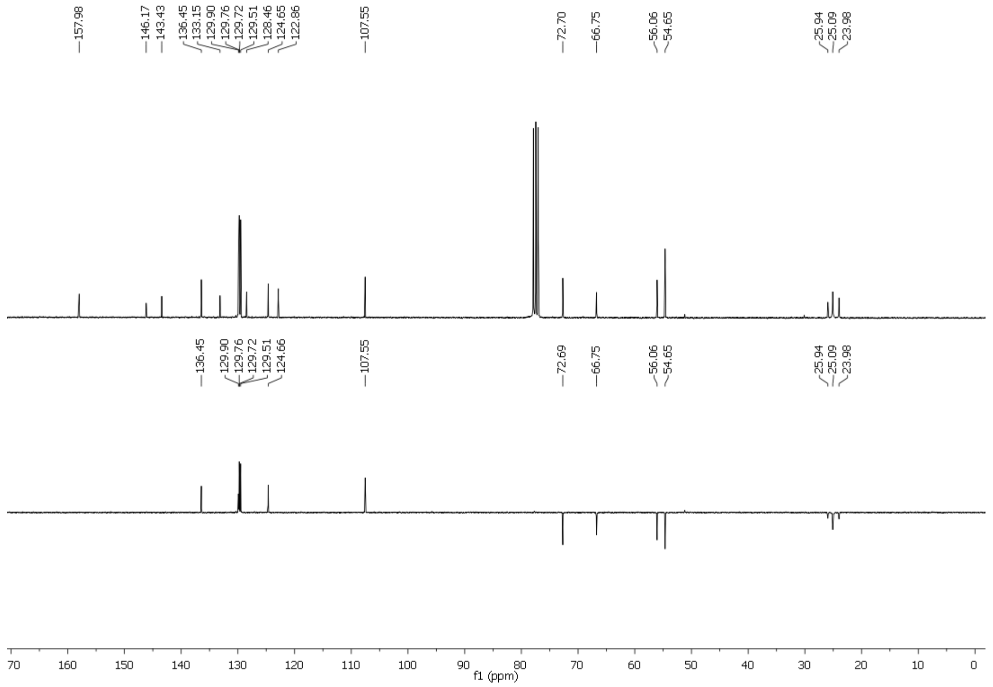

HSQC

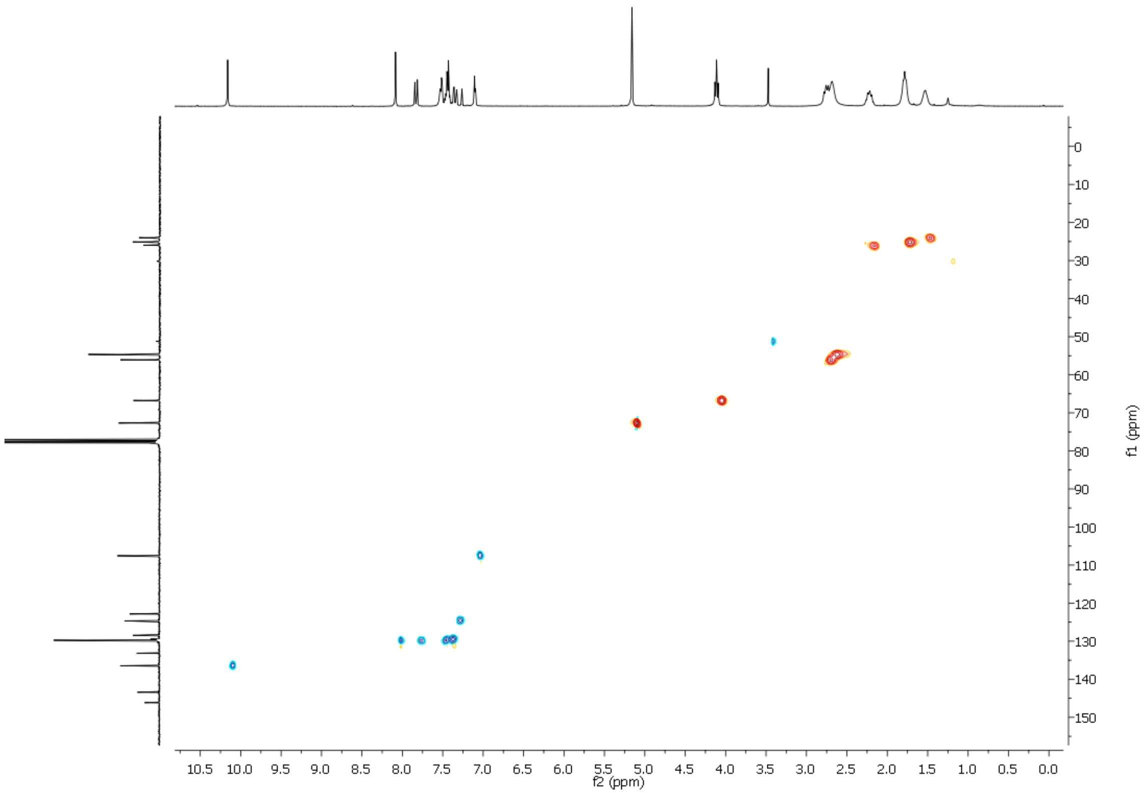

JMA12A

^{1}H NMR

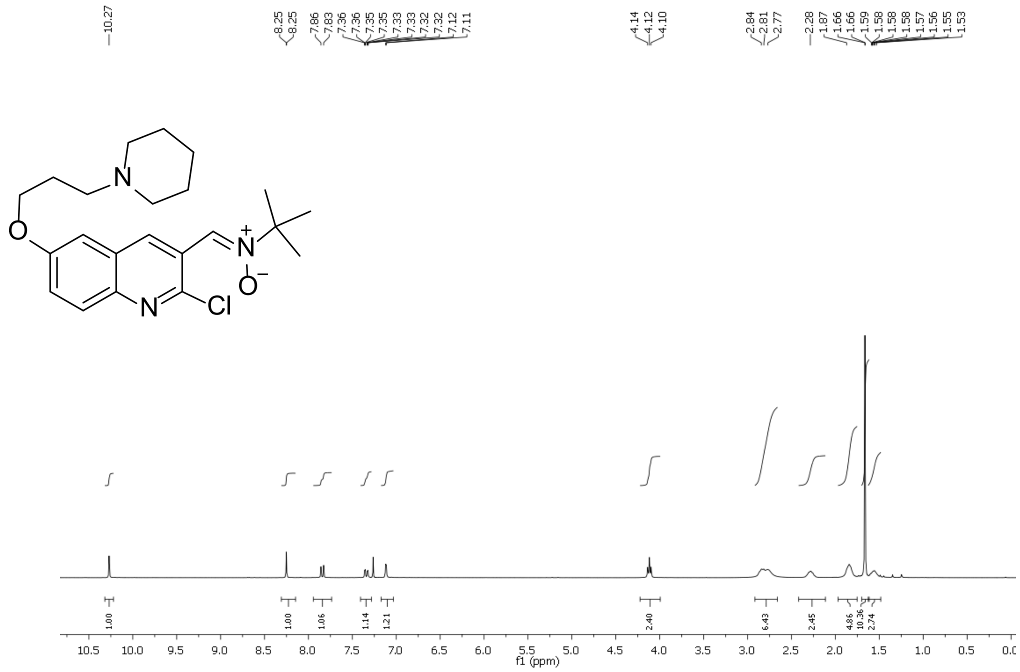

^{13}C NMR

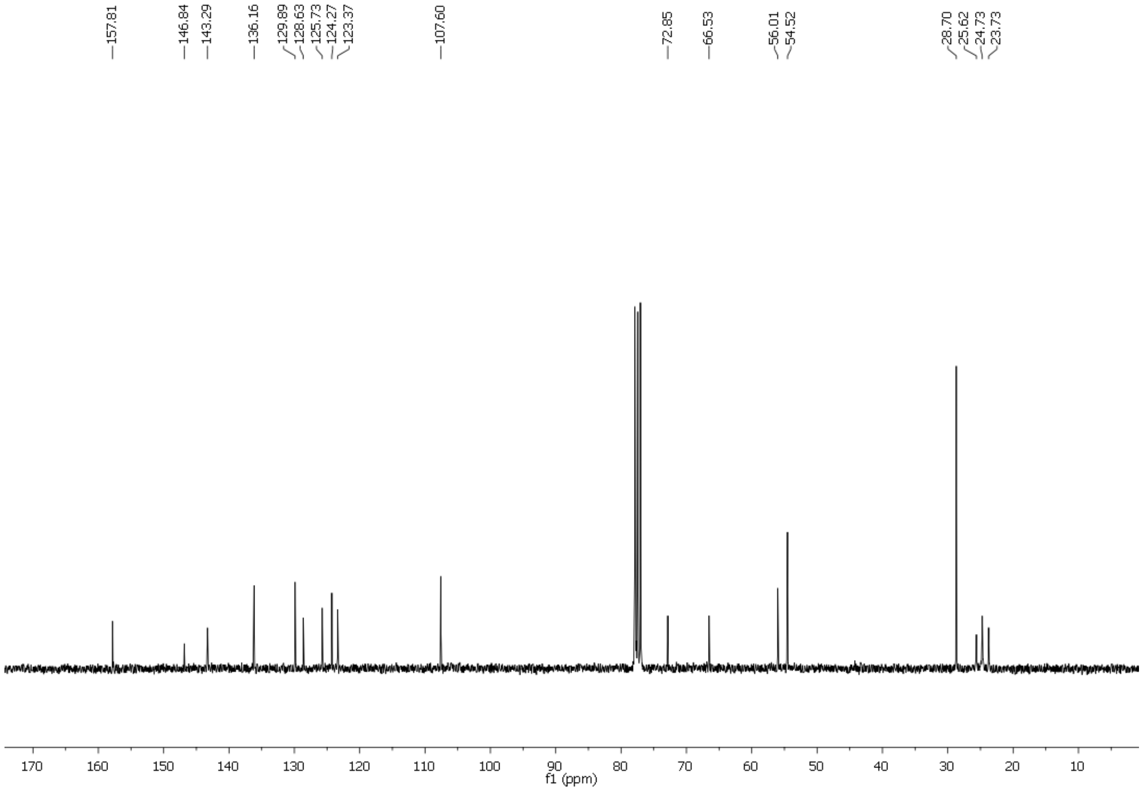

^{13}C NMR vs DEPT

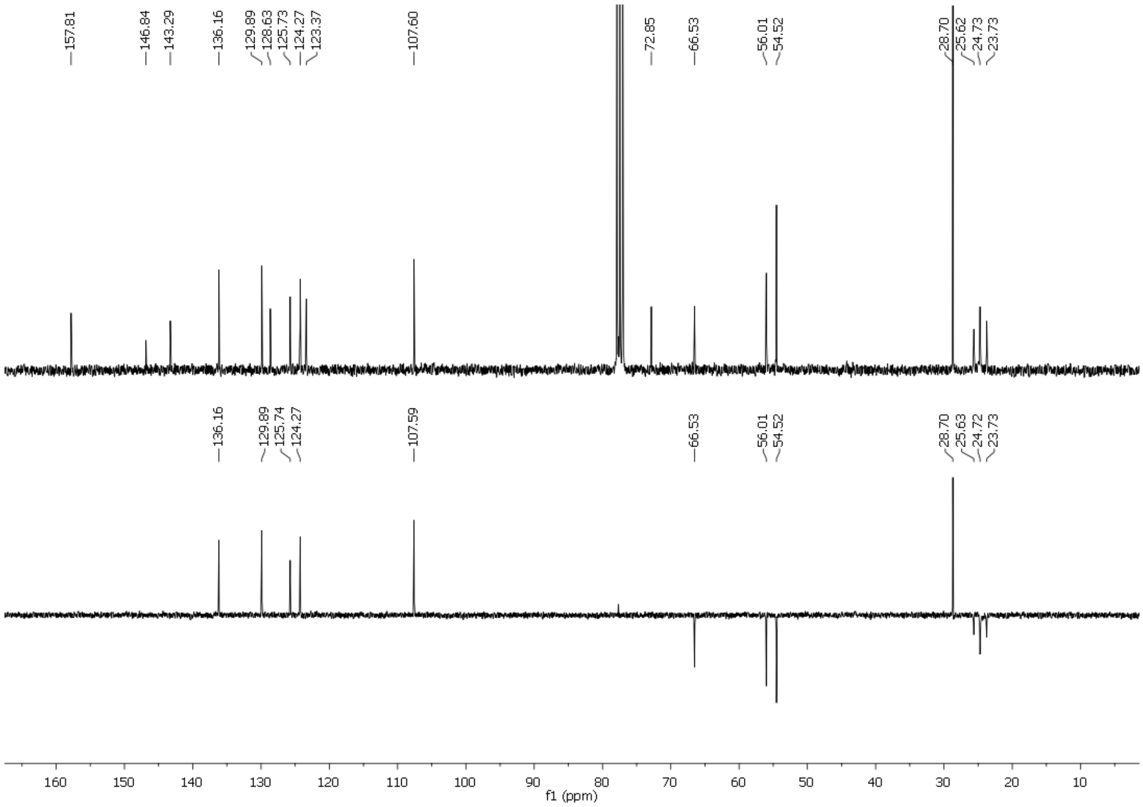

HSQC

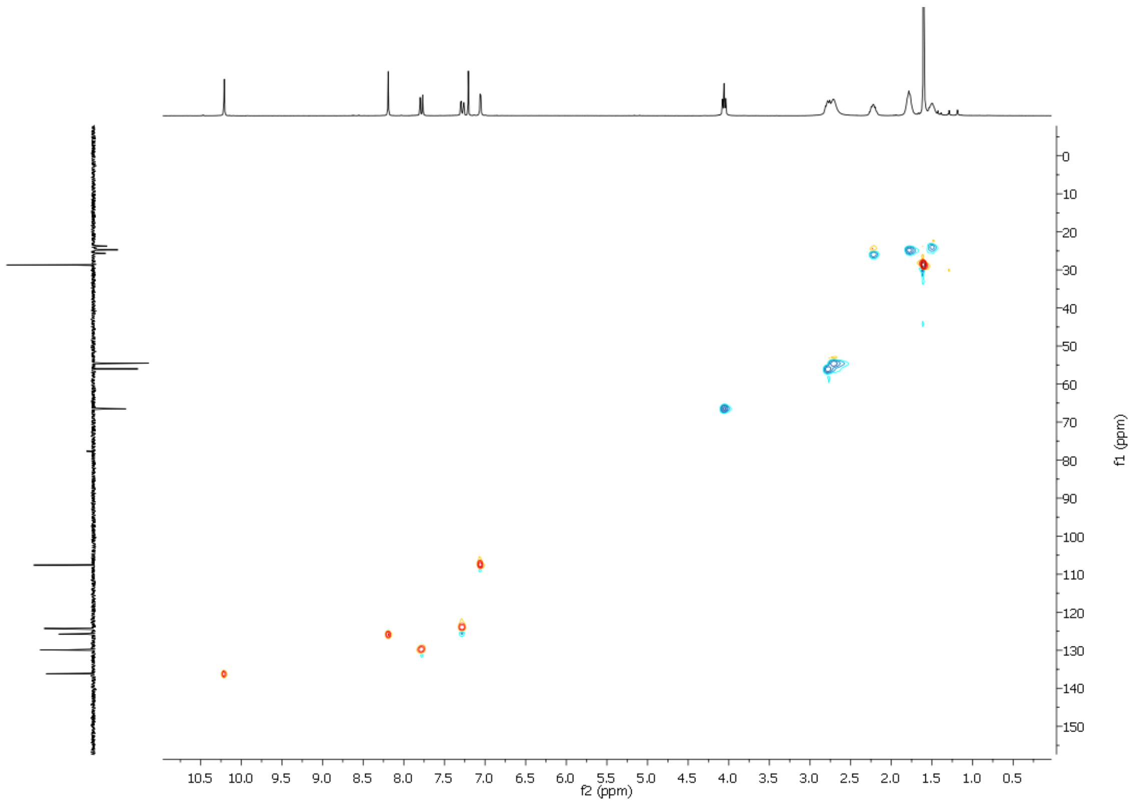

JMA98C

^{1}H NMR

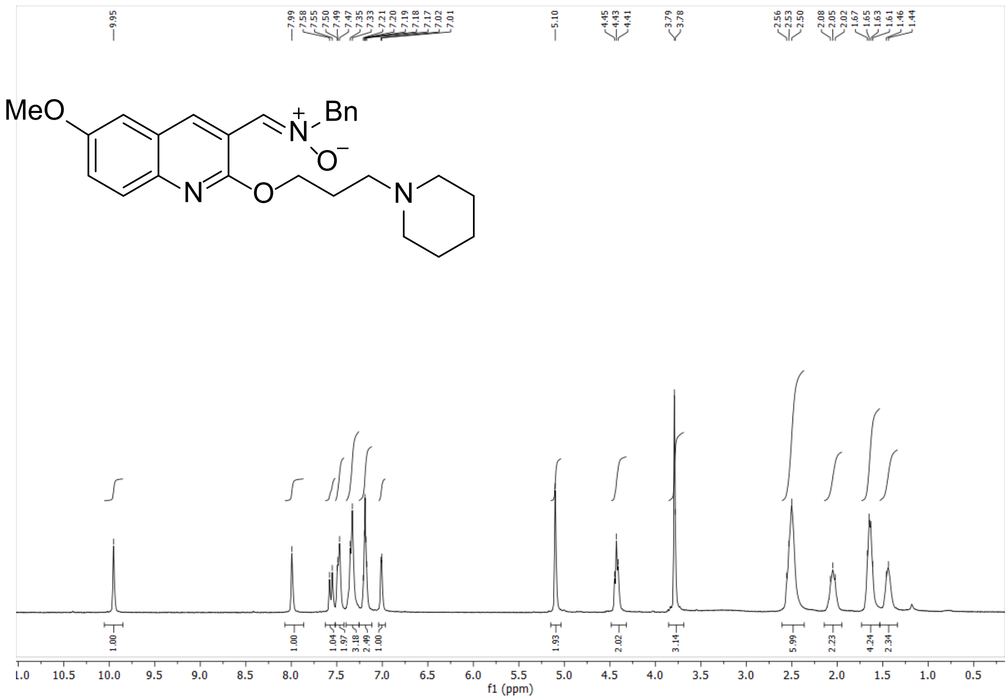

^{13}C NMR vs DEPT

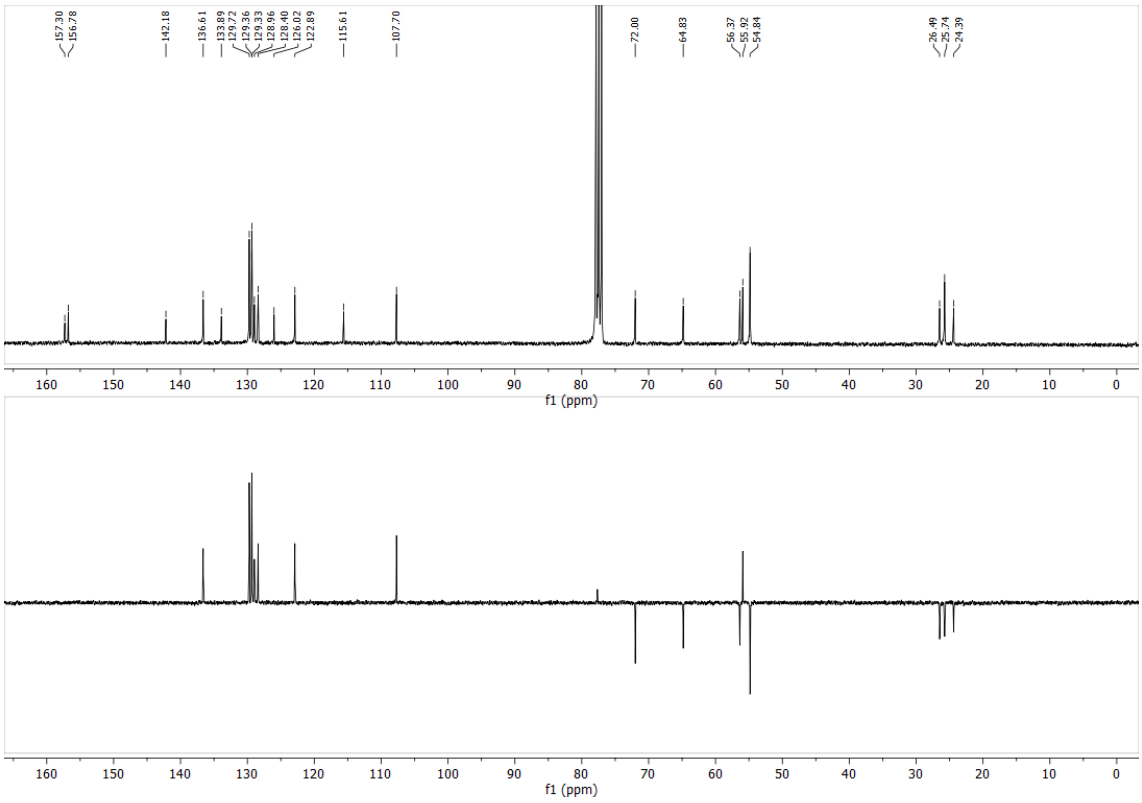

JMA101A

^{1}H NMR

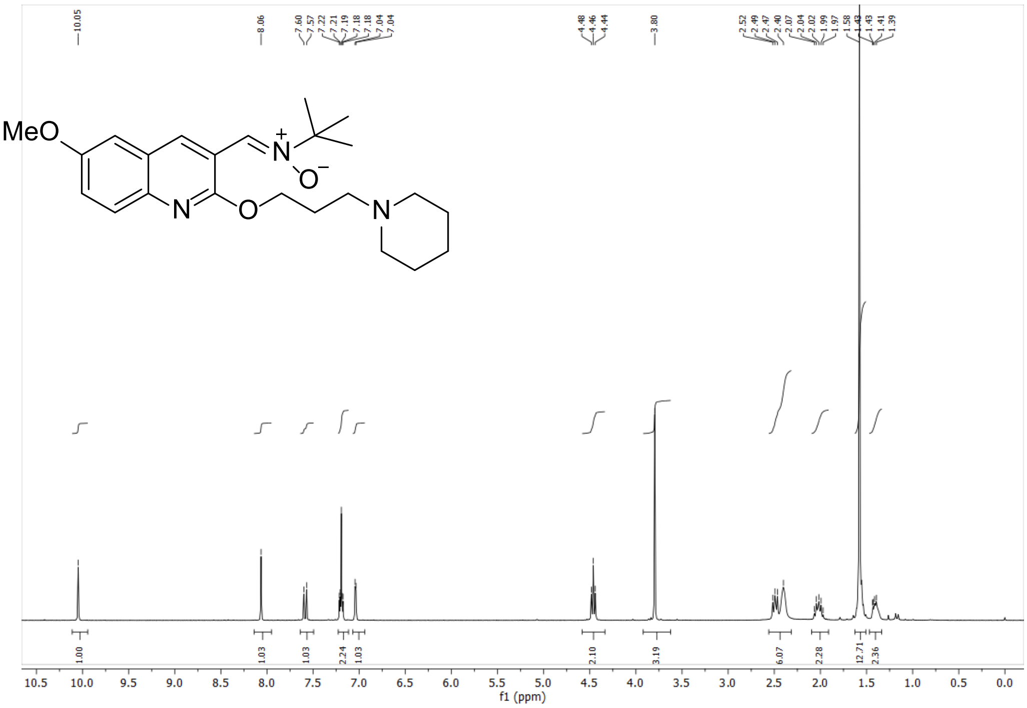

^{13}C NMR vs DEPT

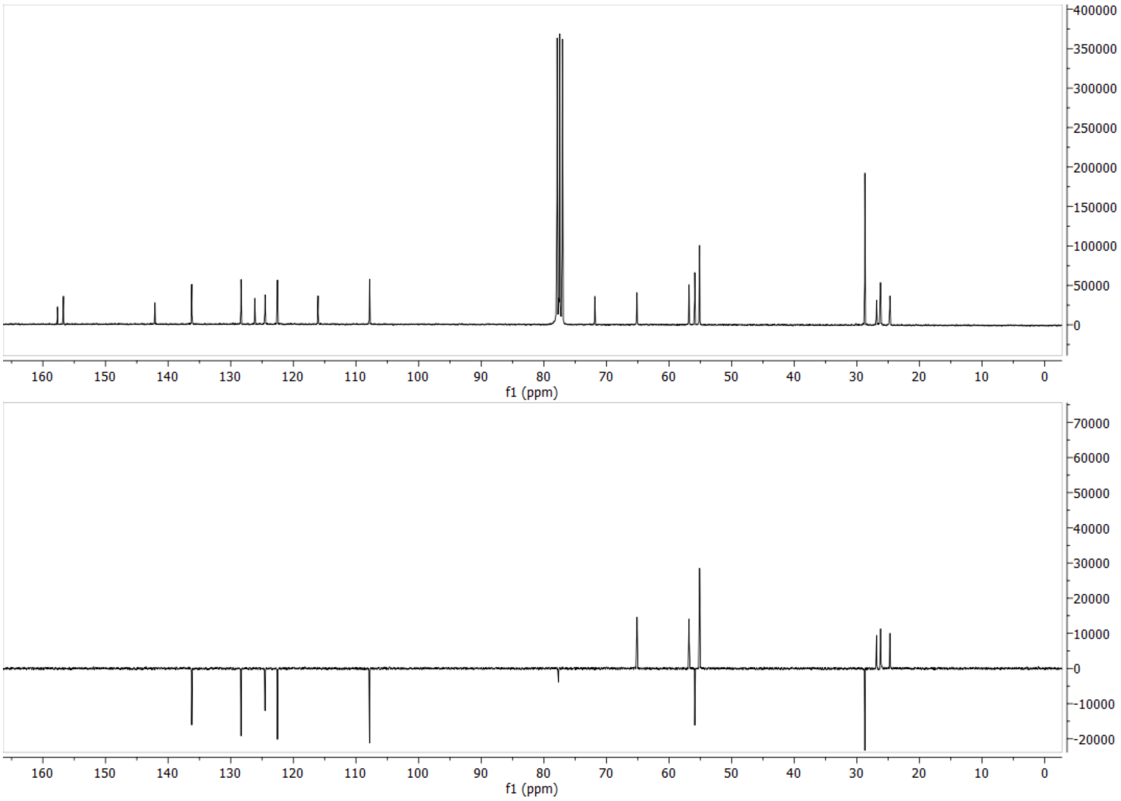

DDI88

^{1}H NMR

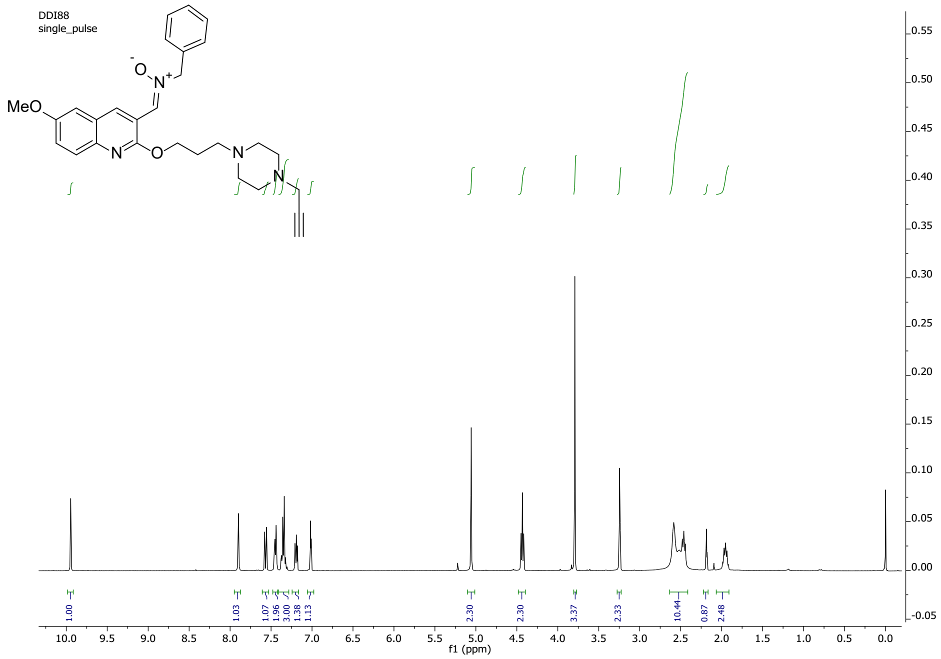

^{13}C NMR

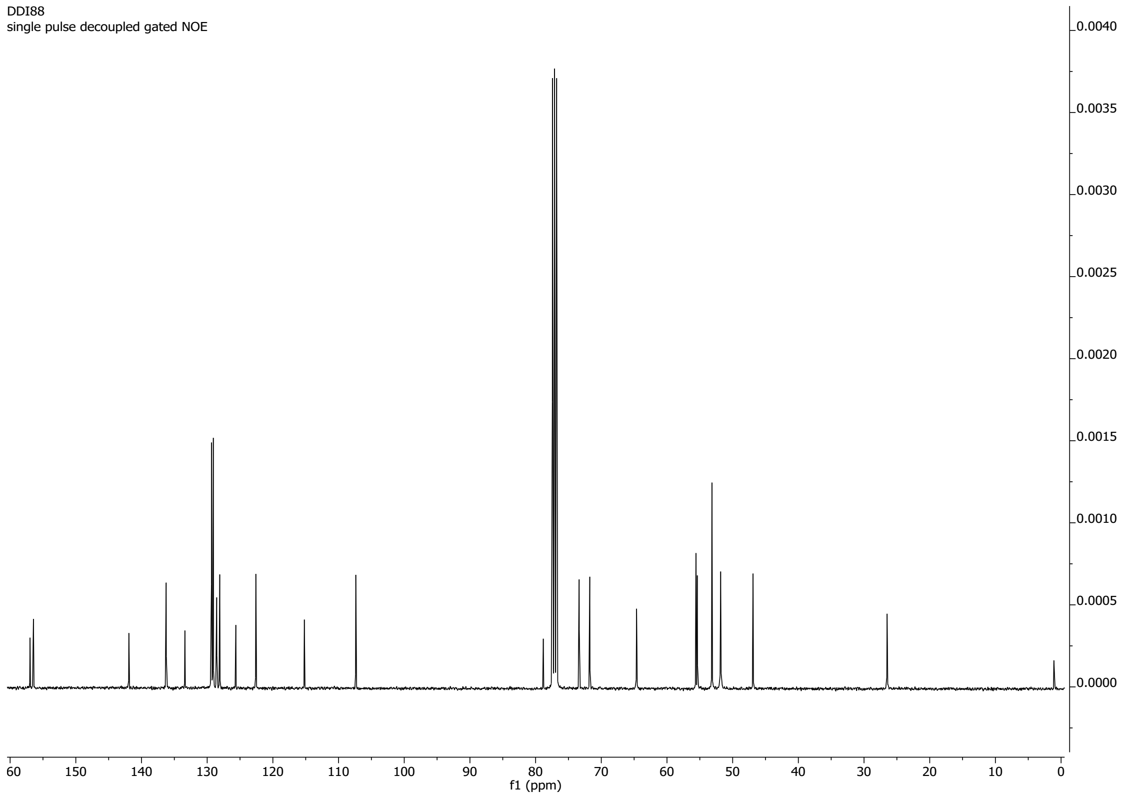

DDI89

^{1}H NMR

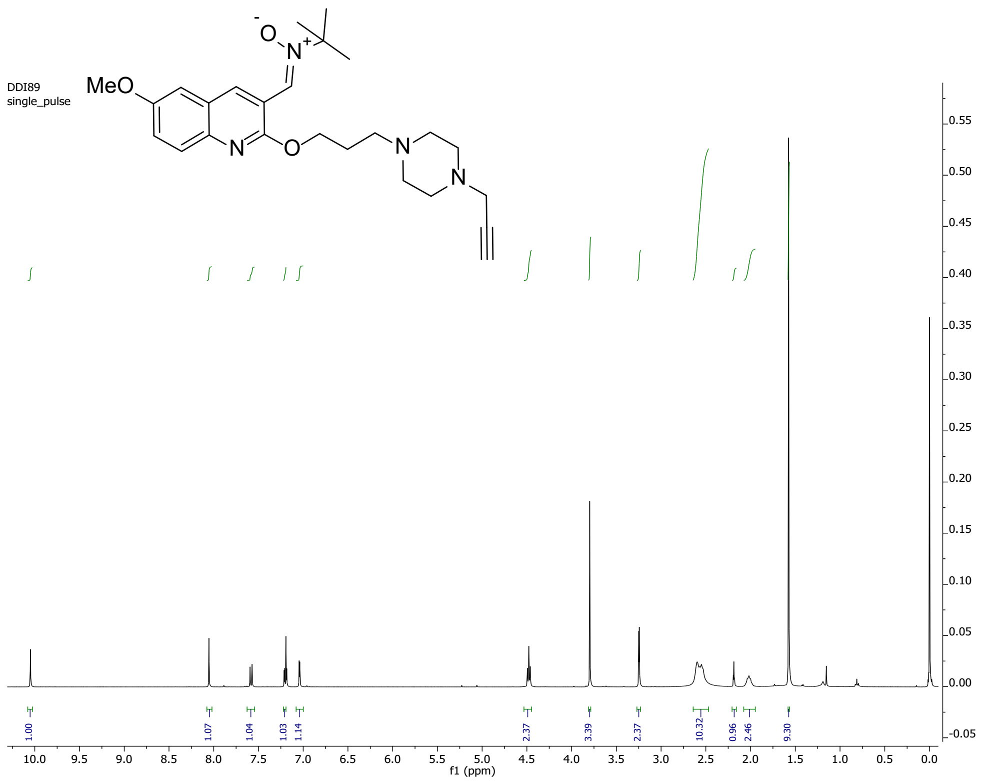

^{13}C NMR

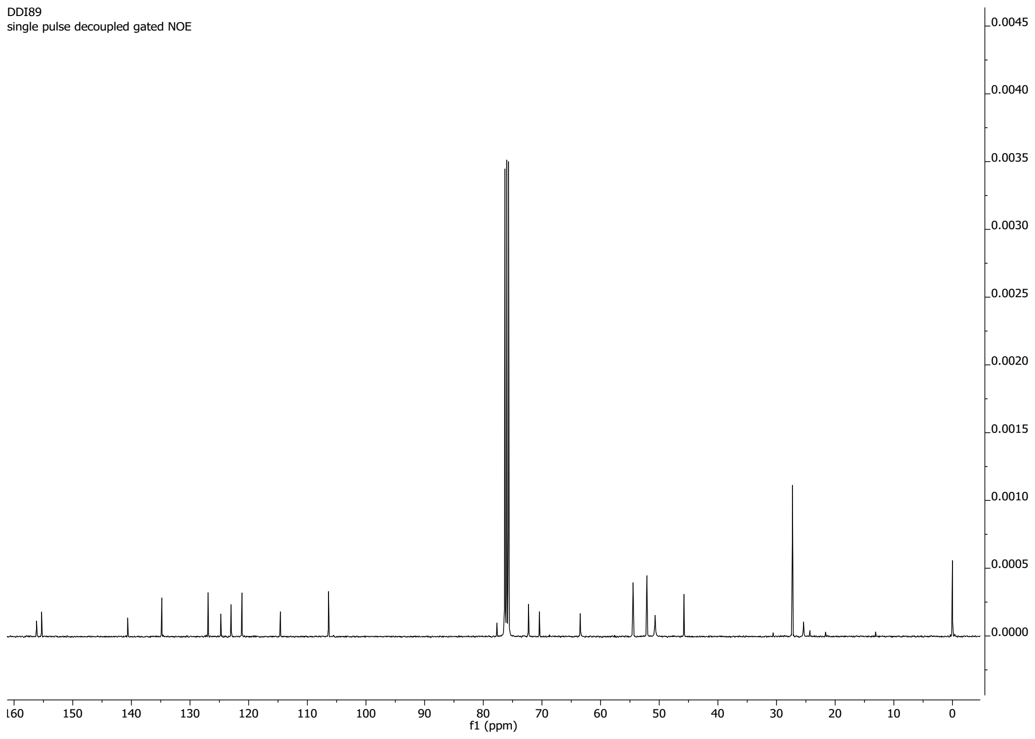

MC902

^{1}H NMR

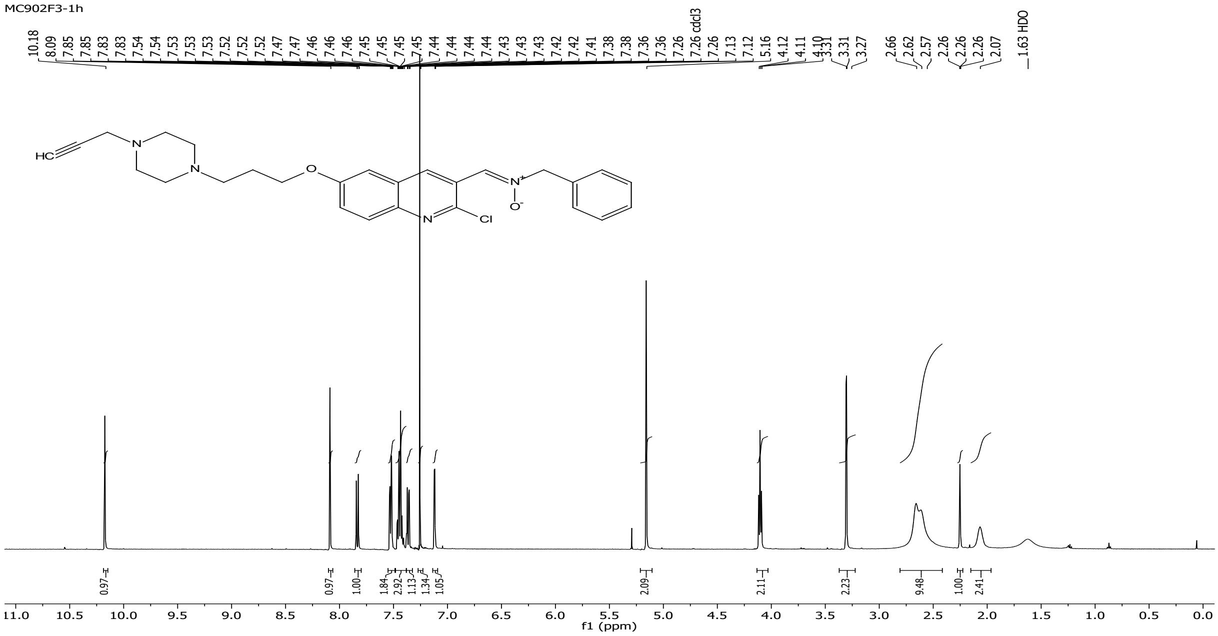

^{13}C NMR

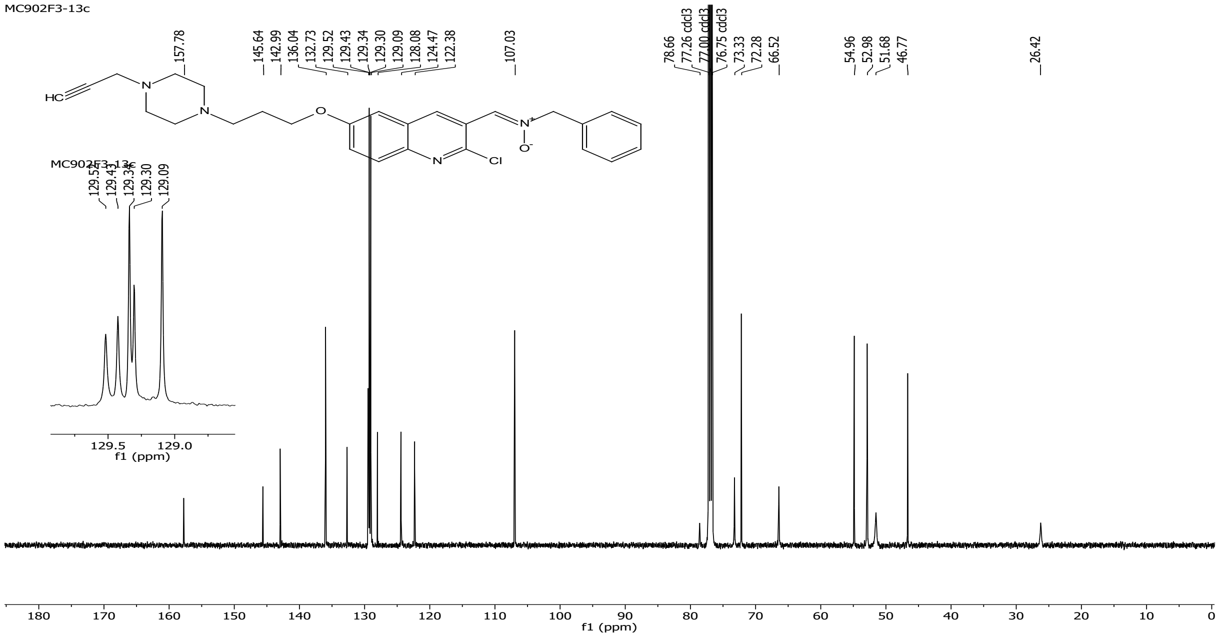

HSQC-NMR

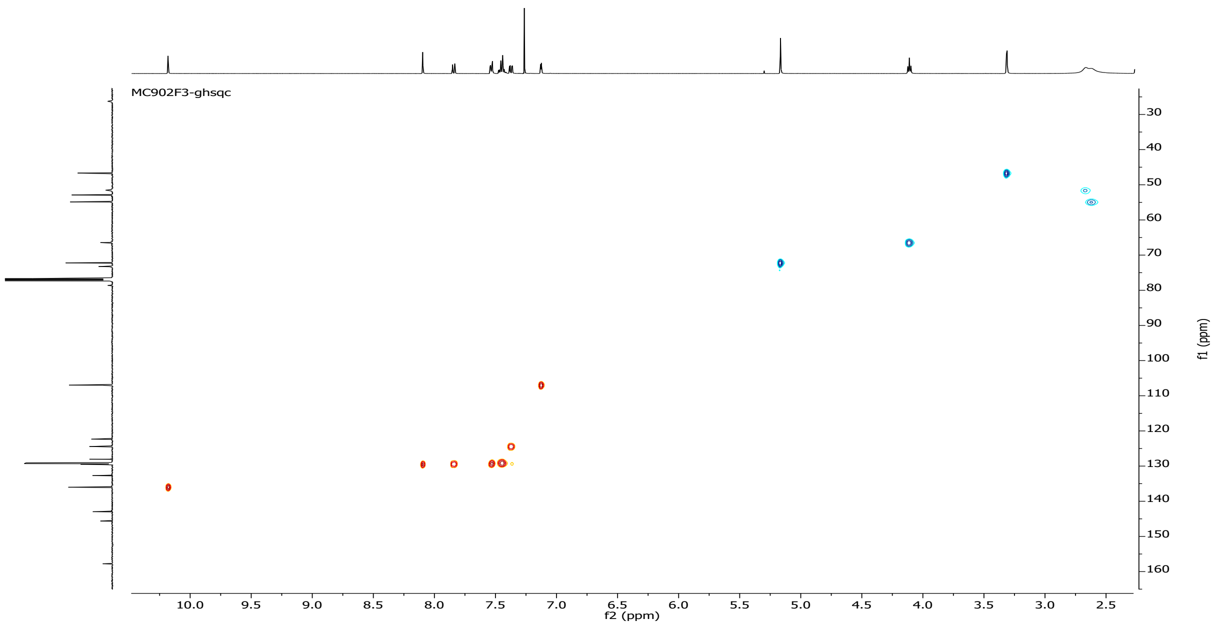

HMBC-NMR

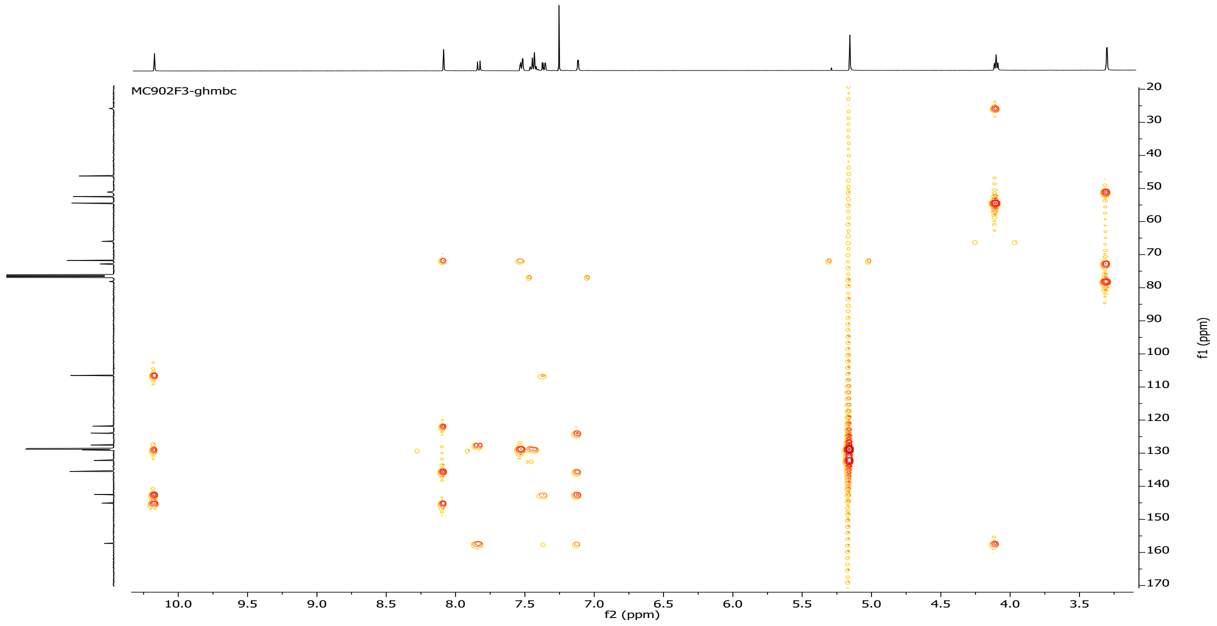

MC903

^{1}H NMR

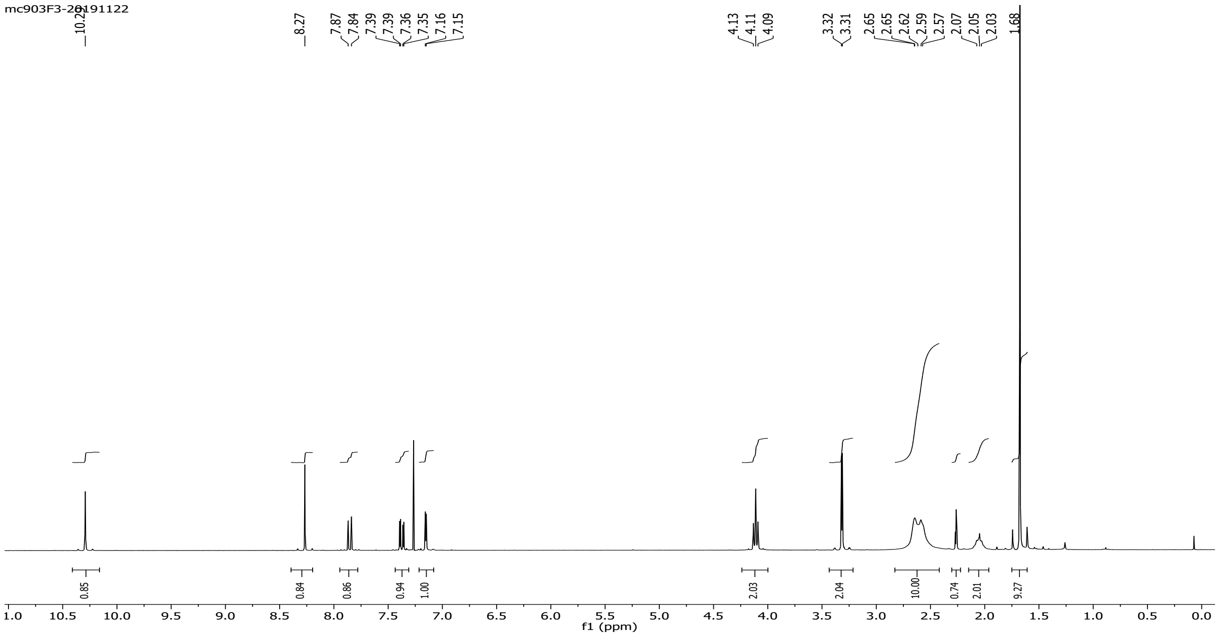

^{13}C NMR

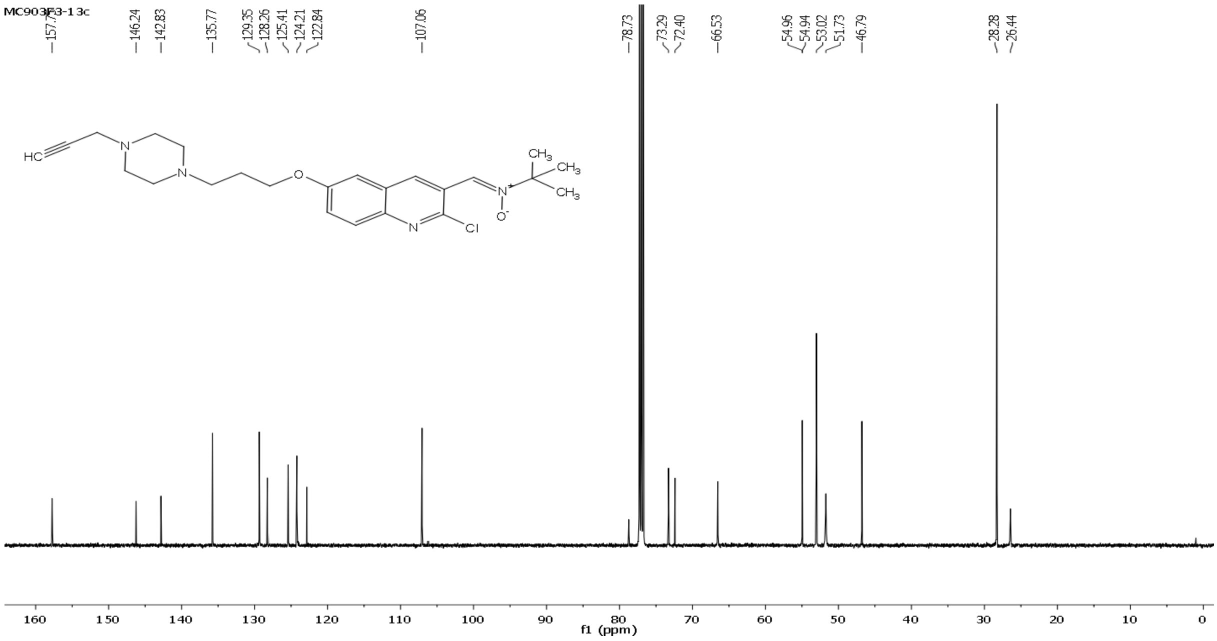

3. Prediction of ADME properties for compound MC903

The expected Absorption, distribution, metabolism, and excretion (ADME) properties of MC903 were evaluated with QikProp module of Schrödinger (QikProp, version 5.1, Schrödinger, LLC, New York, NY, 2017-1). In addition to the ADME properties, drug-likeness properties were also estimated. The drug-likeness of the compound was assessed according to Lipinski's rule of 5 (ROF) and Jorgensen's rule of 3 (ROT). The calculated ADME parameters are presented in Table 1S. It can be seen in Table 1S that all of the parameters are within the reference ranges and this compound has no violation for ROF and ROT.

The partition coefficient (QPlogPo/w), and water solubility (QPlogS) are critical for estimation of absorption and distribution of drug within the body. The values of these parameters for compound MC903 were 4.584 and -4.258, respectively, which were also in the acceptable range.

The significant feature of central nervous system (CNS) active drugs is dependent upon their ability to cross the Blood Brain Barrier (BBB) and display CNS activity. QP log BB is the predicted brain/blood partition coefficient calculated by QikProp and indicates compound BBB permeability, which must be within the range of −3.0 to 1.2. Specifically, the higher the QP log BB value, the greater the ability of the compound to pass through the BBB. QP log BB value of compound MC903 was found to be within the designated range, thus implying the target compound has the potential to penetrate the CNS. Polar Surface Area (PSA) is another crucial predictor for BBB permeability. Molecules with PSA <100 $Å^2$ are more likely to penetrate the BBB and the most active CNS drugs have PSA lower than 70 $Å^2$. The value of PSA for compound MC903 is 49.208 $Å^2$ confirming its BBB permeability potential. Along with QP log BB, apparent Caco-2 (QPPCaco) permeability is considered a vital parameter to predict a compound's distribution within the human body. Importantly, compound MC903 exhibited high permeability in Caco-2 cells.

Further, the prediction for human serum albumin binding using QPlogKhsa, shows that the value for the inhibitor lies within the expected range (-1.5 to 1.5).

The % oral drug absorption predicted for the test compound was adequate with a high percentage (> 96%) of Human Oral Absorption, indicating their possibilities in oral drug formulation.

Overall, compound MC903 possess the appropriate pharmacokinetic profiles required for distribution in the human body and a high probability of being able to successfully penetrate the BBB.

Table S1. Physicochemical properties for compound MC903 calculated using Qikprop

MW	SASA	volume	donorHB	accptHB	QPlogPo/w	QPlogS
442.987	792.690	1439.317	0.500	6.750	4.584	-4.258

QPPCaco	PSA	QPlogBB	metab	QPlogKhsa	% HOA	ROF	ROT
243.916	49.208	0.414	4	0.735	96.512	0	0

MW: Molecular weight of the molecule (130.0-725.0). SASA: Total Solvent Accessible Surface Area, in square angstroms, using a probe with a 1.4 Å radius (limits 300.0-1000.0). volume: Total solvent-accessible volume, in cubic angstroms, using a probe with a 1.4 Å radius (limits 500.0-2000.0). donorHB: Estimated number of hydrogen bonds that would be accepted by the solute (limits: 0.0-6.0). accptHB: Estimated number of hydrogen bonds that would be donated by the solute (limits: 2.0-20.0). QPlogPo/w: Predicted octanol/water partition coefficient (limits -2.0-6.5). QPlogS: Predicted aqueous solubility. S, in mol/dm^3, is the concentration of the solute's saturated solution that is in equilibrium with crystalline solid (limits -6.5-0.5). QPPCaco: Predicted apparent Caco-2 cell permeability in nm/sec. Caco-2 cells is a model for the gut-blood barrier. QikProp predictions are for non-active transport. (< 25 poor, > 500 great). PSA: Van der Waals surface area of polar nitrogen and oxygen atoms (limits 7.0-200.0). QPlog BB: Predicted brain/blood partition coefficient (limits -3.0–1.2). metab: Number of likely metabolic reactions (limits 1-8). QPlogKhsa: Prediction of binding to human serum albumin (limits -1.5-1.5). HOA: Predicted qualitative Human Oral Absorption on 0 to 100% scale. ROF: Number of violations of Lipinski's Rule Of Five (molecular weight < 500, QPlogPo/w < 5, number of hydrogen bond donor ≤ 5, number of hydrogen bond acceptors HB ≤ 10). ROT: Number of violations of Jorgensen's Rule Of Three (QPlogS> -5.7, QPCaco> 22 nm/s, number of primary metabolites < 7).
